# Endocrine and metabolic alterations in response to systemic inflammation and sepsis: a review article

**DOI:** 10.1186/s10020-025-01074-z

**Published:** 2025-01-21

**Authors:** Syed Faizan Mehdi, Muhammad Hamza Qureshi, Salman Pervaiz, Karishma Kumari, Edwin Saji, Mahnoor Shah, Ahmad Abdullah, Kamran Zahoor, Hafiza Amna Qadeer, Disha Kumari Katari, Christine Metz, Lopa Mishra, Derek LeRoith, Kevin Tracey, Michael J. Brownstein, Jesse Roth

**Affiliations:** 1 The Feinstein Institutes for Medical Research/Northwell Health, Manhasset, NY USA; 2 Division of Endocrinology, Diabetes & Bone Disease, Icahn School of Medicine at Mt. Sinai, New York, NY USA; 3 Azevan Pharmaceuticals Inc., Bethlehem, PA USA

**Keywords:** Inflammation, Sepsis, Septic shock, Metabolism, Hormones, Endocrine

## Abstract

Severe sepsis is cognate with life threatening multi-organ dysfunction. There is a disturbance in endocrine functions with alterations in several hormonal pathways. It has frequently been linked with dysfunction in the hypothalamic pituitary-adrenal axis (HPA). Increased cortisol or cortisolemia is evident throughout the acute phase, along with changes in the hypothalamic pituitary thyroid (HPT) axis, growth hormone-IGF-1 axis, insulin-glucose axis, leptin, catecholamines, renin angiotensin aldosterone axis, ghrelin, glucagon, hypothalamic pituitary gonadal (HGA) axis, and fibroblast growth factor-21. These changes and metabolic alterations constitute the overall response to infection in sepsis. Further research is essential to look into the hormonal changes that occur during sepsis, not only to understand their potential relevance in therapy but also because they may serve as prognostic indicators.

## Introduction

Sepsis is one of the oldest recognized medical illnesses or syndromes. It was first mentioned 2700 years ago in Homer’s poems. Hippocrates viewed sepsis as a process during which the body decays, decomposes, and has a foul smell (Funk et al. 2009). It was recognized by Galen as well. (Thurston 2000) Following the establishment of the germ theory by Semmelsweis, Pasteur, and others, sepsis was thought to be due to pathogenic microorganisms that spread through the bloodstream (Thurston 2000; Costa 2002). After antibiotics were developed, clinicians wondered how the drugs could eradicate microorganisms but leave patients vulnerable to sepsis. This suggested that the problem might be a response by the host to the pathogens (Morrison and Ryan 1987; Bone 1993; Shepard et al. 1974).

Sepsis is defined as *“life-threatening organ dysfunction caused by a dysregulated host response to infection or a non-infectious dysregulated inflammatory response,”* according to the most recent guidelines outlined in 2016–The Third International Consensus Definitions for Sepsis and Septic Shock often referred to as Sepsis-3 (Singer et al. 2016). Compared to earlier definitions, this latest one emphasizes systemic inflammation and organ dysfunction. The systemic inflammatory reaction which accompanies sepsis may eventually lead to a vicious cycle of multi-organ failure. Besides affecting various systems, it also leads to disturbances in hormonal function, secretion and metabolic pathways, further aggravating homeostasis (Singer et al. 2016; Wasyluk and Zwolak 2021). Thus, the aim of this review is to summarize our present understanding of alterations in the endocrine system in sepsis and their influence on metabolism (Funk et al. 2009; Khardori and Castillo 2012).

The Phoenix criteria for pediatric sepsis and septic shock, published JAMA 2024, was verified by the International Pediatric Sepsis Definition Task Group utilizing worldwide or international data sets, diverse meta-analysis, systematic literature review, and updated Delphi agreement or consensus strategy. This guideline, or the criteria, uses the Phoenix sepsis score to diagnose sepsis, which is specific by having a score of 2 or more in a patient with suspected or confirmed infection. Septic shock, on the other hand, is described as sepsis with a minimum of one point for cardiovascular on the phoenix score. The score measures organ malfunction throughout four major organ systems like central nervous system, cardiovascular, respiratory and coagulation pathway, which expand to eight in the Phoenix- 8 scoring index with addition to hormonal, kidney, immunological and hepatic systems. In contrast to the earlier specifications or criteria, Phoenix criteria provide much greater sensitivity and specificity, have been extensively evaluated in a multitude of different conditions and situations and exclude SIRS as clinical or diagnostic factor, lowering complication and confusions. This criteria as a tool has been designed to enhance clinical management, improve epidemiological evaluation and clinical research associated with pediatric sepsis and septic shock (Schlapbach et al. 2024).

## Epidemiology

Hospitalizations due to sepsis number more than 970,000 per year and continue to increase at an alarming rate. A 20-year review of data from different hospitals in the US showed an 8.7% yearly surge in the incidence of sepsis (Angus et al. 2001).

Sepsis is the culprit in more than 50% of hospital-related deaths. The mortality rate is directly proportional to sepsis severity: 10–20% of deaths are due to mild-to-moderate sepsis, 20–40% are due to severe sepsis, and 40–48% are due to septic shock syndrome (Angus et al. 2001; Martin et al. 2003; Liu et al. 2014; Martin 2012; Alberti et al. 2002). According to Heffner et al., about 55% of the patients with sepsis-like symptoms who presented to emergency departments had negative blood cultures. No clear infectious source was found in 33% of patients. Thus, patients presenting with sepsis-like symptoms may not always be suffering from an infection. Among conditions like this are pancreatitis, adrenal insufficiency, toxic shock syndrome (bacterial toxins released from Streptococcus or Staphylococcus), trauma, severe burns, and ischemia–reperfusion injuries (Heffner et al. 2010; Marik and Bellomo 2013).

Thyroid dysfunction is among the most common endocrine problems associated with sepsis. This results in a condition known as euthyroid sick syndrome (ESS) or non-thyroidal illness (NTI). ESS patients may initially have normal thyroid-stimulating hormone (TSH) levels and decreased or low triiodothyronine (T3) and there are changes in de-iodination and increase in rT3 levels. (Berghe 2014), and it can develop in people with no history of thyroid disease. The prevalence of ESS in sepsis ranges from 20 to 70% of cases (Via et al. 2024), and lab values can vary based on the length and severity of sepsis. Inadequate adrenal function, or adrenal insufficiency, is seen in one-third (20 to 30%) of the patients with septic shock (Annane et al. 2002). Stress hyperglycemia in patients without any history of diabetes has also been noted. Recent studies have shown that 20–50% of non-diabetic patients have stress hyperglycemia when they are septic (Berghe et al. 2001a).

## Pathophysiology

Uncontrolled inflammation in sepsis results in an increase in the release of pro-inflammatory cytokines, including tumor necrosis factor alpha (TNF-α), interleukin-1 beta (IL1-β), IL-6, and HMGB-1. These cytokines activate neutrophils as well as macrophages, triggering a state of systemic inflammation and compromising endocrine and metabolic homeostasis (Hotchkiss et al. 2016; Angus and Poll 2013; Schulte et al. 2013; Galley 2011).

Activation of the complement system causes endocrine dysfunction. C5a levels are elevated in sepsis as a result of complement activation, leading to complement mediated activation of neutrophils and macrophages. This releases reactive oxygen species (ROS) and further impairs immune function. (Guo and Ward 2005) Damage associated molecular patterns (DAMPs) are present inside the cell in physiological states that are exposed to the immune system in cases of stress and inflammation. Among them, high mobility group box 1 (HMGB1) is released by necrotic tissues in inflammatory conditions such as sepsis (Chen and Núñez 2010). HMGB1 reacts with toll like receptors, leading to endocrine dysfunction causing insulin resistance and impaired glucose metabolism during sepsis (Klune et al. 2008).

Disrupted thyroid function is another condition caused by a dysregulated immune response and can lead to euthyroid sick syndrome. The patients suffering from this condition have low triiodothyronine (T3) levels despite having normal thyroid-stimulating hormone (TSH) levels. This disruption in thyroid function further causes metabolic derangements and exacerbates the catabolic state observed in septic patients (Zhang et al. 2024).

## Hormonal axis dynamics in acute and chronic sepsis

The autonomic, immune, and endocrine systems interact with one another to execute the body's response to sepsis and other inflammatory conditions. The foreign threat is first recognized by the immune system. The immune system then communicates with the central nervous system (CNS) through cytokines, leading to the activation of neurohormonal axes. It should be noted that endocrine changes are different in acute and chronic sepsis (Gheorghiţă et al. 2015). In acute sepsis, the body over-activates the sympathetic adreno-medullary system and the hypothalamus–pituitary–adrenal axis, causing a catecholamine and cortisol surge. As a result, blood glucose levels increase, energy stores are mobilized, and immune responses are modulated. Activation of vasopressin and the renin–angiotensin–aldosterone system (RAAS) promotes salt and water retention, thereby regulating blood pressure and volume. These processes redirect the body's resources to the stressed areas of the body and inhibit nonessential physiological functions (Gheorghiţă et al. 2015).

During chronic sepsis, which can last from hours to days, the body’s hormonal profile changes significantly. Some of the major changes that can occur are related to the body’s inability to keep up with the constant state of stress it has been subjected to for a prolonged period of time. One such change is disruption of the body's circadian rhythm. This is followed by a decrease in vasopressin, adrenal non-responsiveness to adrenocorticotropic hormone (ACTH), and disruption in thyroid functions, leading to 'euthyroid sick syndrome (Berghe 1998a; Schuetz and Müller 2006; Boonen and Berghe 2016).

Along with being associated with higher mortality rates, elevated catecholamine levels are known to have adverse effects on the gut. Due to redirection of resources to the vital areas of the body, continuous catecholamine surges lead to decreased blood flow and weakened immune responses in the gut. The decreased blood flow shifts the body’s energy source from glucose to fatty acids. It also promotes increased virulence and bacterial proliferation, along with an increase in blood clotting. (Andreis and Singer 2016) (Table 1).

**Table 1:** Endocrine-induced effects and immune changes during the acute and established phase of sepsis

Hormone	Endocrine changes	Immune changes
Cortisol (Arafah 2006; Marik et al. 2008)	Acute: ↑	Innate: ↑↓ immune function including cell differentiation, phagocytosis and cytokine release
	Established: ↑↓ with loss of diurnal variation. Often ↓ response to exogenous stimulation	Adaptive: ↓ lymphocyte activation but ↑ apoptosis; ↓ cytokines and chemokines; ↑ Th2 and Treg cell expression over Th1 and Th17 cells
Catecholamines (Hartmann et al. 2017; Rudiger and Singer 2016; Elenkov 2007; Lyte 1992; Hahn et al. 1995)	Acute: ↑	↓ Gut immunity; ↑ bacterial growth and virulence; ↑ immune suppression
	Established: ↑↓ but increased hypo-responsiveness	Innate: α-ARs activation ↑ inflammation; β2-AR activation ↓ inflammation including chemotaxis, phagocytosis, and ROS for respiratory burst
		Adaptive: β2-AR activation ↓T-cell proliferation but ↑Th2 polarization
Thyroid hormones (Lee and Farwell 2016; Fliers et al. 1997)	Acute: ↑ but soon after ↓TSH; ↓T4 to T3 conversion; ↓T3; ↑rT3 (sick euthyroid syndrome)	Innate: ↑↓ chemotaxis, phagocytosis and respiratory burst. Sick euthyroid syndrome ↓ immune function; ↑ monocyte differentiation to DCs rather than macrophages
	Established: TSH normalizes but loses pulsatility; ↓TRH; ↓T3	Adaptive: ↑↓ lymphocyte proliferation and apoptosis, and B-cell antibody production
Insulin (Agwunobi et al. 2000; Read and Cheng 2007; Wang et al. 2006)	Acute: ↓	Innate: ↓ respiratory burst and NET formation in neutrophils; ↓ proinflammatory cytokines
	Established: ↑ but also ↑insulin resistance	Adaptive: ↑ lymphoid cell lineage expression; ↑ T-cell proliferation, differentiation, and effector functions
Glucagon (Michie 1996; Bessey et al. 1984)	Acute: ↑	Innate: ↓ chemotaxis and respiratory burst; ↑↓ neutrophil numbers
	Established: ↑	Adaptive: ↓ T-cell proliferation, differentiation, and effector functions
Leptin (Arnalich et al. 1999)	Acute: ↑	Innate: ↑ cytotoxicity of NK cells; ↑ activation of granulocytes, DCs, and macrophages
	Established: ↓	Adaptive: ↓ T-cell proliferation and responsiveness; ↓ Th cell differentiation; ↑ Treg cell proliferation; ↓ B-cell proliferation but ↑ apoptosis (Zhou et al. 2021)

## Hypothalamic pituitary adrenal (HPA) axis

The hypothalamus produces corticotropin-releasing hormone (CRH), which stimulates the pituitary to secrete adrenocorticotropic releasing hormone (ACTH). ACTH stimulates the adrenal cortex to produce cortisol. Increased cortisol in the blood, in turn, inhibits the hypothalamus and the pituitary through negative feedback. (Wasyluk et al. 2021) Under normal conditions, there is a circadian rhythm in cortisol release. This rhythm is lost in sepsis and other stressful conditions, which are associated with increased ACTH secretion. (Cooper and Stewart 2003) Pro-inflammatory cytokines, including IL-5, IL-6, and TNF-α released in sepsis, cross the blood–brain barrier and act on the hypothalamus and the pituitary to stimulate CRH and ACTH release, respectively. This leads to an increase in baseline cortisol levels in the blood (Turnbull and Rivier 1999; Annane et al. 2017). In chronic sepsis, desensitization occurs and there is a decrease in cortisol release (Cooper and Stewart 2003).

In addition to increased cortisol production, alterations in glucocorticoid (GC) metabolism contribute to the elevated cortisol levels seen in critically ill patients (Boonen et al. 2013). Cortisol is primarily metabolized in the liver via A-ring reductases and in the kidneys via 11β-hydroxysteroid dehydrogenase type 2. It is thought that elevated circulating bile acid levels impede the action of cortisol metabolizing enzymes, thus increasing cortisol levels. This is reflected in the increase in half-life of cortisol observed in severely ill patients (Boonen et al. 2013; Boonen et al. 2014 Apr 15; Vanwijngaerden et al. 2011). There is also a reduction in cortisol-binding globulin (CBG) in severe sepsis due to an increase in neutrophil elastase in the blood. The decrease in CBG is associated with a reduction in cortisol binding and an increase in free cortisol in the serum. (Wasyluk and Zwolak 2021).

Even though sepsis is characterized by hypercortisolemia, increased production of cortisol is transient, and secretion of ACTH, the main cortisol stimulant, tends to normalize. The elevated cortisol in the bloodstream initiates the negative feedback through the central glucocorticoid receptor alpha (GR-α) (Téblick et al. 2021; Téblick et al. 2022). A study done on C57BL/6 mice showed a reduction in the gene expression of prohormone convertase 1/3 (PC 1/3), the enzyme responsible for conversion of the prohormone pro-opiomelanocortin (POMC) to its active form ACTH, indicating decreased ACTH production. Additionally, Annexin A1, an anti-inflammatory mediator of glucocorticoids known to regulate feedback at the pituitary level via its suppressive effect, was found to have increased expression in the same mouse models (Téblick et al. 2021).

**Fig. 1:**
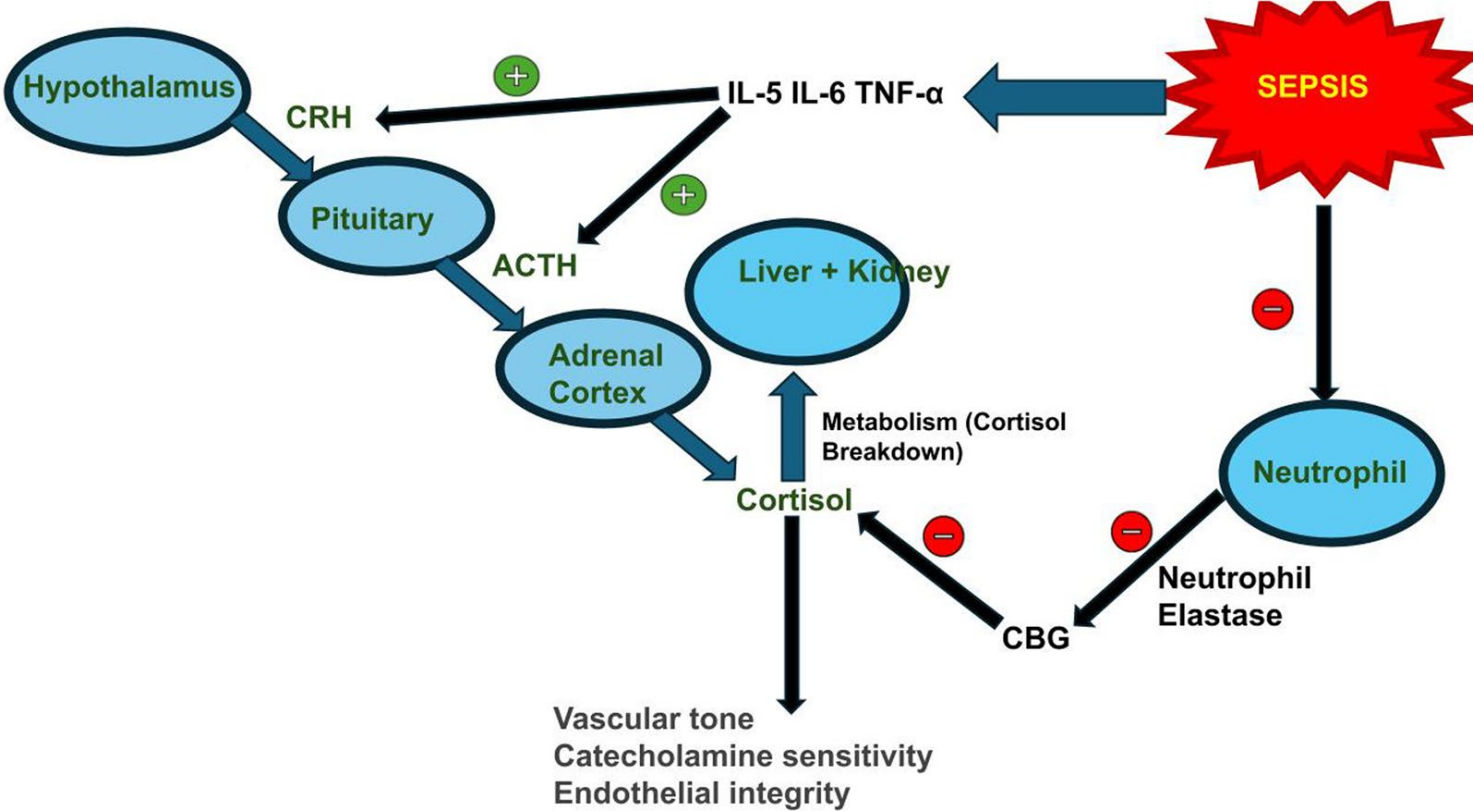
HPA-Axis in Acute Sepsis: (Original figure) The hypothalamus releases CRH, which stimulates pituitary ACTH secretion. This leads to cortisol release from the adrenal cortex. Pro-inflammatory cytokines released during sepsis stimulate CRH and ACTH secretion promoting hypercortisolemia. Cortisol maintains vascular tone, catecholamine sensitivity, and endothelial integrity. *ACTH* Adrenocorticotropic Hormone, *CRH* Corticotropin-releasing Hormone, *IL-5* Interleukin-5, *IL-6* Interleukin-6, *TNF-α* Tumor Necrosis Factor-Alpha

Increased cortisol concentrations have beneficial as well as harmful effects on the body. The beneficial effects of increased cortisol include anti-inflammatory effects, maintenance of vascular tone, catecholamine sensitivity, and endothelial integrity. In contrast, the deleterious effects of long-term cortisol increases include an increased likelihood of infections and the development of myopathies. Cortisol also drives hyperglycemia. Hyperglycemia can provide a rapid source of energy for stressed cells in sepsis, but it has harmful effects on the body in the long run (Arafah 2006). The above description of HPA axis responses in sepsis outlines what is typically encountered, but a condition called critical illness-related corticosteroid insufficiency (CIRCI) is also seen. This condition is defined as “inadequate cellular corticosteroid activity for the severity of the patient’s illness” (Marik et al. 2008; Liang et al. 2021). It can either be caused by endocrine cell damage, dysregulation of the HPA axis, altered cortisol metabolism, or tissue resistance to GCs. (Annane et al. 2017) (Fig. 1).

## Hypothalamic pituitary thyroid (HPT) axis

Thyroid hormones play a key role in body growth, cell differentiation, and metabolism. The secretion of thyroid hormone is under the influence of thyroid stimulating hormone (TSH) secreted by the pituitary, which, in turn, is stimulated by secretion of thyrotropin-releasing hormone by the hypothalamus. Thyroid hormone consists of a less active thyroxine (T4) and a more active triiodothyronine (T3). Most of the thyroid hormone released is T4, which is converted into T3 and its inactive isoform, reverse triiodothyronine (rT3), by deiodinase enzymes in peripheral tissues (Yen 2001). This equilibrium is disturbed in states of stress. When patients develop a critical illness such as sepsis, the very first response is a sudden drop in T3 levels, accompanied by a simultaneous rise in rT3 levels. TSH and T4 levels vary widely from person to person. (Michalaki et al. 2001; Peeters et al. 2003; Yildizdaş et al. 2004; Rodriguez-Perez et al. 2008; Yanni et al. 2019; Hong et al. 2024) Most commonly, there is a decrease in T3, an increase in rT3, and no significant change in TSH. This is called euthyroid sick syndrome (ESS) (Lee and Farwell 2016).

The initial drop in T3 in the acute phase is caused by a block in type 1 deiodinase, the enzyme that catalyzes the conversion of T4 to T3 in the periphery (Lee and Farwell 2016). This is a compensatory response to stress leading to reduced energy consumption, inhibition of protein catabolism, and a negative effect on ROS (Selvaraj et al. 2008; Langouche et al. 2013; Mebis et al. 2012). Other factors thought to alter thyroid hormone levels in sepsis include concentrations of thyroid binding globulins (TBG), inhibition of the nuclear thyroid hormone receptor, and changes in free fatty acids and bilirubin, which affect hormone transport and metabolism (Lim et al. 1993; Brinker et al. 2005).

In contrast to what is seen in the acute phase, the major changes observed in chronic sepsis include a drop in T4, TSH, and T3. This drop is caused by central adaptations, characterized by decreased expression of TRH in the hypothalamus (Fliers et al. 1997). The exact cause of the central dysregulation is still unclear. Cytokines, neuropeptide Y, hypercortisolemia, changes in the activity of hypothalamic deiodinase, and thyroid hormone transporter expression have all been identified as potential causes of the problem. Moreover, iatrogenic causes, such as glucocorticoid administration, may also contribute to this condition (Ingels et al. 2018).

Initially, it was thought that changes in thyroid hormone concentrations in sepsis were attributable to structural alterations in thyroid follicular cells due to apoptosis. Apoptosis causes cell death, but subsequent loss of follicular cells can indirectly, over time, change the thyroid cell population profile during sepsis. A study was done by Lin et al., who induced sepsis in male Sprague Dawley rats by cecal ligation and puncture and took blood samples from the right common carotid artery at 12 h, 24 h, and 36 h after induction. They observed a gradual increase in caspase 3, an indicator of apoptosis, while the concentration of both T3 and T4 decreased gradually, with a rapid drop observed at the 36-h mark. They also observed the thyroid follicular cells under a transmission electron microscope at different times over the subsequent 48 h after cecal ligation and puncture and found ultrastructural alterations in the thyroid follicles (Lin et al. 2016).

**Fig. 2:**
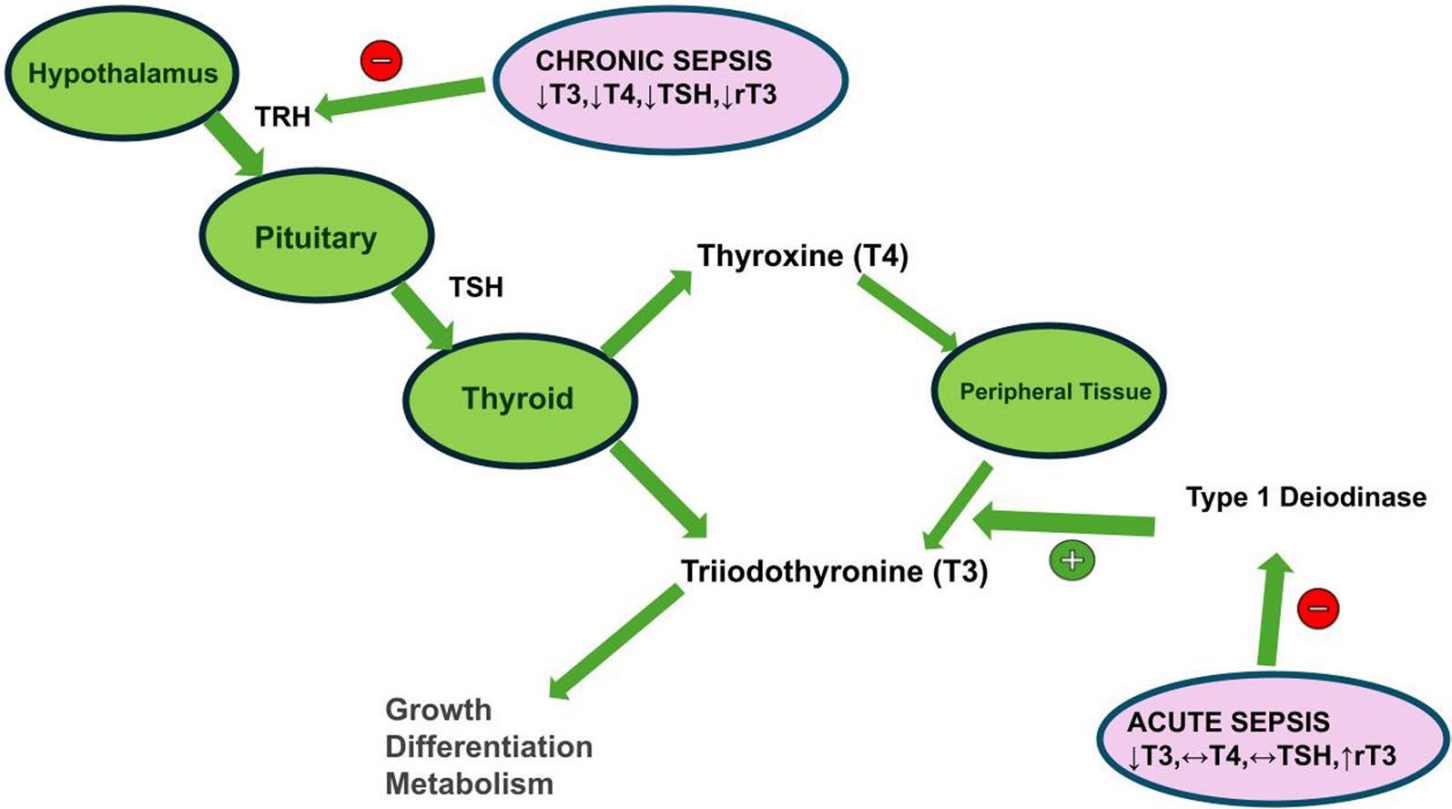
HPT-Axis in Acute and Chronic Sepsis. (Original figure) The hypothalamus releases TRH which stimulates TSH secretion leading the thyroid gland release of active T3 and less active T4 (which is converted to T3 in the periphery). Cytokines and other factors acting in acute sepsis mainly affect the type 1 deiodinase responsible for converting T4 to T3 in the peripheral tissue, decreasing T3 while T4 and TSH remain the same and rT3 is increased. In chronic sepsis, the central HPT-axis is affected causing a decrease in T3, T4, TSH, and rT3. Legend: *TRH* Thyrotropin-releasing Hormone, *TSH* Thyroid Stimulating Hormone, *T4* Thyroxine, *T3* Triiodothyronine, *rT3* Reverse Triiodothyronine

Changes in the hypothalamus-pituitary-thyroid (HPT) axis have been shown to be correlated with mortality of critically ill patients (Foks et al. 2019). Kothiwale et al. looked at the correlation between the Acute Physiology and Chronic Health Evaluation II (APACHE II) score of sepsis patients admitted to the ICU and thyroid hormone levels. They found that the APACHE II score was negatively correlated with functional T3 (fT3) and functional T4 (fT4) and positively correlated with TSH (Kothiwale et al. 2018) (Fig. 2).

## Growth hormone or IGF-1 axis

Pulsatile release of growth hormone (GH) from the anterior pituitary is stimulated by hypothalamic growth hormone releasing hormone (GHRH) and inhibited by somatostatin (Giustina and Veldhuis 1998). GH acts directly on target organs, having a predominantly catabolic effect–increased lipolysis and glycogen breakdown–or indirectly through insulin-like growth factor 1 (IGF-1) which has anabolic actions including glucose uptake, decreased lipid breakdown, and protein synthesis (Berneis and Keller 1996).

In acute sepsis, IL-1, IL-6, and TNF-α decrease the expression of hepatic GH receptors, suppressing IGF-1 and IGF-binding protein 3 (IGFBP-3) production. This leads to the development of GH resistance and elevated GH levels, promoting the catabolic effects of GH (Timmins et al. 1996; Bentham et al. 1993). However, this increase is transient and tends to shift as sepsis progresses. In the chronic phase, GH secretion tends to decrease as well. This is thought to cause the wasting syndrome seen in some chronically septic patients (Onenli-Mungan et al. 2004).

GH concentrations have been observed to be positively correlated with sepsis severity and mortality; greater GH levels have been observed in patients with septic shock as opposed to sepsis. On the other hand, IGF-1 levels are negatively correlated with sepsis severity. IGF-1 is lower in septic shock than in mild to moderate sepsis (Groof et al. 2002; Papastathi et al. 2013; Berghe 2002).

## Insulin-glucagon-glucose axis

### A. insulin

Agwunobi et al. administered lipopolysaccharide injections to induce endotoxemia and observed hypoglycemia within the first 2 h after administration of LPS and the development of hyperglycemia afterwards (Agwunobi et al. 2000; Read and Cheng 2007). Hyperglycemia is thought to be due to the development of insulin resistance secondary to the actions of counter-regulatory hormones, i.e., growth hormone, cortisol, and catecholamines. In addition, TNF-α, one of the first proinflammatory cytokines secreted in sepsis, has been shown to promote insulin resistance. (Taylor and Beilman 2005; Desborough 2000).

Initially, treating septic patients with insulin was thought to decrease severity, with significant reductions in mortality ranging from 7 to 11%. It also reduced septicemia, mechanical ventilation time, organ failure, and polyneuropathy (Song et al. 2014; Berghe et al. 2003, 2006; Ellger et al. 2008; Vlasselaers et al. 2009). These results have not been confirmed in more recent studies, however. The Normoglycemia in Intensive Care EvaluationᅳSurvival Using Glucose Algorithm Regulation (NICE-SUGAR) T rial reported lower mortality rates with comparatively lenient glucose control, regardless of the patients' diabetic status. (Finfer, et al. 2009) A trial conducted by Costantini et al. to determine the benefits of strict glucose control had to be stopped early because of an increased rate of hypoglycemic events. (Costantini et al. 2021) Furthermore, Yamada et al. emphasized the lack of any clinical benefit of intensive insulin therapy in terms of mortality. There were increased rates of hypoglycemia in both diabetic and non-diabetic patients on tight glucose control as compared to patients on mild (140–180 mg/dl) and very mild control (180–220 mg) (Yamada et al. 2017). Current guidelines recommend treating hyperglycemia in critical patients to a target of 140–180 mg/dL, no matter whether the people are diabetic or not (Rhodes et al. 2017; American Diabetes Association 2020).

**Fig. 3:**
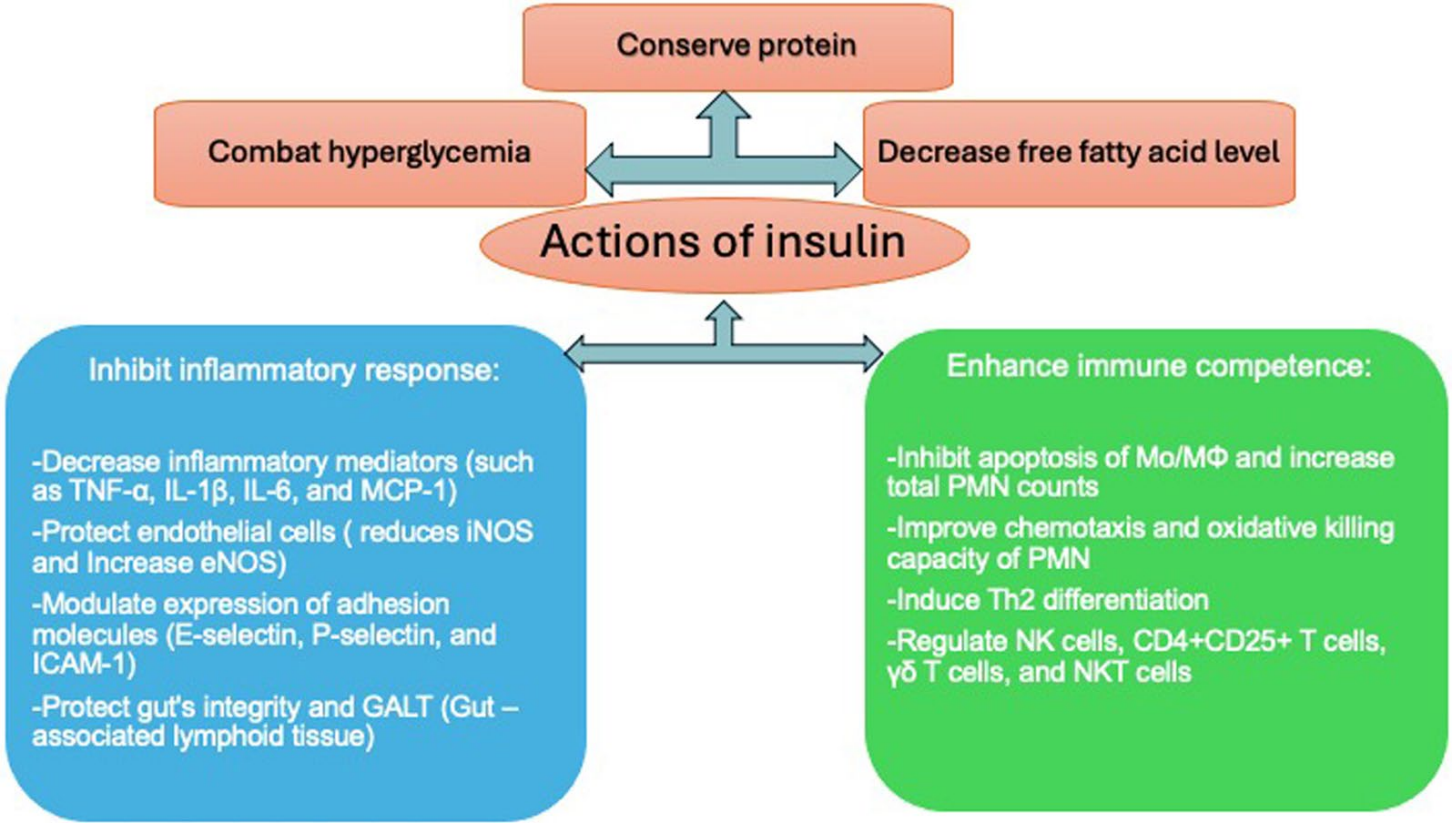
Actions of Insulin: Proinflammatory and Anti-inflammatory responses Figure modified from Open access (International Immunopharmacology) permissible to re-use under a CC-BY 4.0 license)

In addition to glucose regulation, insulin is also responsible for inflammatory and immune response modulation (Jeschke et al. 2002, 2004, 2004; Chang et al. 2021; Berghe 2004). Insulin balances the effects of pro-inflammatory cytokines (IL-1ß, IL-6) vs anti-inflammatory cytokines (IL-10). (Jeschke et al. 2005) It downregulates acute-phase proteins, decreases complement levels, and increases endothelial nitric oxide (Wang et al. 2006; Hansen et al. 2003; Takebayashi et al. 2004; Zhao et al. 2007a). In trauma, systemic inflammatory response syndrome (SIRS), or severe burns, insulin reduces pro-inflammatory mediators (TNF-a, IL-6, CRP, IFN-y, IL-18) (Wang et al. 2007, 2005; Zhao et al. 2007b) (Fig. 3).

### B. insulin resistance

Studies of glucose metabolism in individuals given lipopolysaccharide (LPS) injections to replicate sepsis have revealed a two-phase reaction. There is an initial surge in insulin sensitivity within 2 h of giving LPS, followed by the emergence of insulin resistance after several hours. This suggests that the initial rise in peripheral and hepatic insulin sensitivity, leading to hypoglycemia, is primarily fueled by heightened glucose disposal, and reduced endogenous glucose production. The subsequent phase of insulin resistance, occurring approximately 7 h post-LPS administration in humans and as early as 3 to 5 h afterwards in animals given higher LPS doses, is not well understood. (Agwunobi et al. 2000; Crabben et al. 2009; Virkamaki and Yki-Jarvinen 1994).

A number of mechanisms have been proposed to account for insulin resistance in sepsis: suppression of serine phosphorylation resulting from glucocorticoid-induced increases in fatty acids, blockade of PI-3 K pathways by proinflammatory cytokines, and reduction in insulin-facilitated translocation of GLUT-4 in the plasma membrane of cardiac and skeletal muscle caused by activation of adenosine monophosphate-activated protein kinase signaling (Delile et al. 2017; Li and Messina 2009; Vanhorebeek et al. 2005).

In humans, the onset of insulin resistance coincides temporally with the emergence of TNF, a factor that is believed to act in concert with other cytokines. During sepsis, cortisol, growth hormone, and catecholamine concentrations increase, and these agents may also play a role in the development of insulin resistance (Agwunobi et al. 2000).

Insulin resistance has several possible explanations. The potential inhibition of the beta subunit's tyrosine kinase activity by inflammation linked to sepsis can lead to a reduction in the intracellular regulation of glucose, lipid, and protein metabolism (Yaribeygi et al. 2019). Furthermore, the presence of proteolytic activity in the serum can liberate the receptor from the plasma membrane. Research conducted by Bauza-Martinez et al. has revealed that individuals suffering from septic shock exhibit heightened proteolytic activity and display specific plasma peptidomic profiles that correlate with mortality (Bauza-Martinez et al. 2018).

It is plausible that heightened proteolytic activity during the initial inflammatory phase could result in a decrease in the number of insulin receptors on the cell's plasma membrane, thereby contributing to the development of insulin resistance. Under such circumstances, the insulin receptors present in the bloodstream could potentially intercept insulin molecules, thereby impeding their effects on the cells. Furthermore, the process of insulin receptor recycling back to the plasma membrane might be disrupted in cases of sepsis (Chen et al. 2019). Hancock and colleagues have documented the migration of insulin receptors to the cell nucleus, where they have shown that these receptors interact with genetic promoters and influence gene expression (Hancock et al. 2019).

### C. glucagon

Glucagon is a peptide hormone secreted by pancreatic alpha cells in response to hypoglycemia, prolonged fasting, exercise, and protein-rich meals (Gerich et al. 1975). Its release is regulated by endocrine and paracrine pathways, nutritional substances, and the autonomic nervous system (Gromada et al. 2007). It is responsible for the breakdown of fatty acids and amino acids and increasing blood glucose via hepatic glycogenolysis and gluconeogenesis. (Unger 1985; Miller and Birnbaum 2016; Galsgaard et al. 2019; Holst et al. 2017) At high doses, glucagon can also regulate heart rate and contractility (Ceriello et al. 2016).

Sepsis leads to a surge in stress hormones in the blood, including catecholamines, cortisol, growth hormone, and glucagon (Michie 1996). Glucagon is known to play more of a supporting role in the catabolic activities of other hormones. Bessey et al. observed a mild catabolic response with increased metabolic rate, accelerated glucose production, and a net negative nitrogen balance when they administered cortisol to test subjects. However, when they administered cortisol along with glucagon and epinephrine, the catabolic response was significantly increased (Bessey et al. 1984).

Jung et al. were the first to study the effects of glucagon on sepsis exclusively. They followed 112 sepsis patients through their hospital stay and measured their glucagon levels at days 0, 1, 3, and 7, correlating them with the clinical picture. They found that the glucagon levels were positively correlated with the severity of the condition, as determined by the Acute Physiology and Chronic Health Evaluation (APACHE) II and the Sequential Organ Failure Assessment (SOFA) scores. Moreover, glucagon levels also predicted the mortality of the patients studied. The average glucagon level of survivors was 53.0 (40.0–99.5) pg/mL, as compared to 85.0 (50.0–149.0) for non survivors. This trend was seen throughout the course of the study [75.9 ± 7.27 pg/mL versus 109.8 ± 11.52 pg/mL on day 0, 69.09 ± 7.03 versus 99.31 ± 12.24 pg/mL on day 1, 62.18 ± 6.13 versus 98.85 ± 16.55 pg/mL on day 3, and 62.51 ± 5.85 versus 124.85 ± 25.36 pg/mL on day 7] (Jung et al. 2015). Thus, glucagon may be a useful biomarker and deserves to be studied in more detail (Jung et al. 2015).

### D. glucose

During sepsis, hyperglycemia is commonly observed in patients, irrespective of their diabetes status (Umpierrez et al. 2002). Hyperglycemia is mostly due to lipolysis, glycogenolysis, and hepatic gluconeogenesis. In addition, the utilization of muscle lactate to produce glucose (Cori Cycle) in the liver in patients suffering from shock further adds to hyperglycemia in sepsis (Levy 2006).

While studying the proteomic analysis of peripheral blood mononuclear cells (PBMCs) from septic patients, it was observed that proteins related to glycolysis, pentose phosphate pathway (PPP), and the production of ROS were upregulated while the proteins involved in the tricarboxylic acid cycle and oxidative phosphorylation were downregulated as compared to the values in the control group on the day of admission, indicating activation of PPP and ROS production and inhibition of the tricarboxylic acid cycle and oxidative phosphorylation. Seven days post admission, these proteins had almost gone back to their initial levels, but proteins related to hypoxia-inducible factor 1a (HIF-1a) and liver X receptor/retinoid X receptor (LXR/RXR) pathway were upregulated both at admission and 7 days later. Moreover, the enzymatic activity of the electron transport chain (ETC) was impaired as well, suggesting mitochondrial dysfunction. This could be the cause of the increased production of ROS in these patients (Ferreira et al. 2022).

While studying the association between mean blood glucose (MBG), glycemic variability (GV), and ICU mortality in sepsis, Lu et al. found that ICU mortality was strongly influenced by MBG and GV, and their impact increased with the increasing severity of the disease. This was not the case in individuals with certain pre-existing conditions like diabetes, immunosuppression, liver disease, or documented hypoglycemia; no significant relationship was observed in them. The optimal MBG target to minimize ICU mortality was determined to be between 120 and 140 mg/dl for nondiabetic patients. The authors also set an MBG target between 140 and 190 mg/dl for diabetics, but no significant reduction in ICU mortality was observed in these patients (Lu et al. 2022).

Glucose metabolism can influence our bodies’ responses to viral infections as well. For example, hepatitis B virus (HBV) can stimulate hexokinase (HK) and lactate dehydrogenase A (LDHA), increasing glycolysis and lactate production in the cells. Normally, the retinoic acid inducible gene-1 (RIG-1) interacts with mitochondrial antiviral signaling (MAVS) to produce interferons. The excess lactate produced suppresses this interaction, leading to reduced interferon and thus, escaping detection by the immune system (Zhou et al. 2021). Glucose metabolism also regulates interferon regulatory factor 5 (IRF5). IRF5 is responsible for regulating the inflammatory response in a variety of viral infections and autoimmune disorders. In the case of the immune response to COVID-19, the purpose is to address the linkage that connects mitochondrial ROS) with glycolysis, as this may help in averting a dangerous immune response known as a “cytokine storm.” Learning how glucose metabolism affects viral infections will be useful in controlling the immune response and potentially enhancing antiviral therapies (Zhang et al. 2023).

**Fig. 4:**
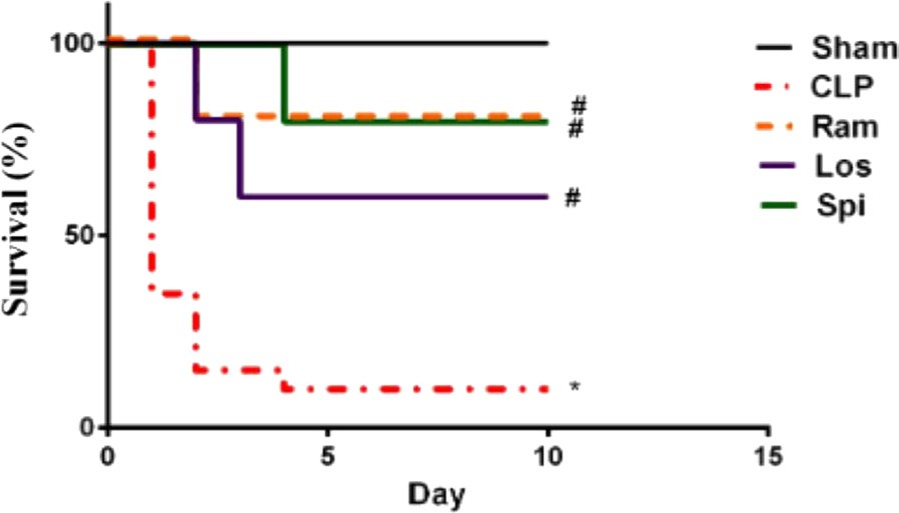
RAAS inhibitors and cecal ligation and puncture. Kaplan Mier survival curves in rats. (n = 10 rats/group except CLP-nontreated group (n = 20 rats) * Statistically Significant difference compared from Sham (p < 0.05), # Statistically Significant difference compared from CLP (p < 0.05). CLP = Cecal ligation and puncture, Spi = Spironolactone, Los = Losartan, Ram = Ramipril. Figure modified from Open access (Fundamental & Clinical Pharmacology) permissible to re-use under a CC-BY 4.0 license)

Influenza virus decreased the expression and activity of G6PD and thus downregulated the expression of NRF2, which regulates the antioxidant response. As a result, oxidative stress and viral replication increase. Influenza virus proteins promote glycolysis by stimulating the Mammalian target of rapamycin complex 1 (mTORC1) and Mammalian target of rapamycin complex 2 (mTORC2) pathways upregulating GLUT1 and HIF-1α expression, respectively. Dengue virus (DENV) activates the PI3K/Akt/mTOR pathway to upregulate GLUT expression, thus enhancing glycolysis. HCMV activates AMPK (Adenosine Monophosphate activated protein kinase) via (Ca2 + /calmodulin-dependent protein kinase kinase) CaMKK. AMPK activation stimulates glycolysis to provide energy and facilitate virus replication. HCMV infections upregulate GLUT4, which increases glucose uptake. Activated AMPK in turn stimulates glycolysis by GLUT4. HBV achieves glycolytic metabolic recombination by increasing GLUT1 expression. HBV regulates HK activity, and LDHA stimulates lactic acid production, thereby inhibiting RIG-I interaction with MAVS and leading to immune escape by regulating IFN production. SARS-CoV-2 activates the PI3K/Akt/mTOR pathway, leading to the release of GP73, which stimulates liver gluconeogenesis enhancing fasting glucose through cAMP/PKA. 2-DG. Metformin inhibits viral replication through the mechanisms of suppressing glycolysis and gluconeogenesis, respectively (Figure 4) (Lu et al. 2022; Zhou et al. 2021; Zhang et al. 2023).

## Somatotrophic axis/ghrelin

Ghrelin is a peptide hormone secreted in the stomach. Its major functions include stimulating appetite and growth hormone secretion (Date et al. 2000). In addition, ghrelin acts on the cardiovascular, gastrointestinal, reproductive, immune, and central nervous systems (Kojima and Kangawa 2005; Delporte 2013; Leite-Moreira and Soares 2007). Treatment with ghrelin has been shown to reduce inflammation and, therefore, reduce disease severity in conditions like sepsis, inflammatory bowel disease, arthritis, pancreatitis, obesity, diabetic nephropathy, and cachexia. It has also shown promising results in experimental autoimmune encephalomyelitis and in several other murine models of autoimmune diseases (Chorny et al. 2008; Granado et al. 2005; Warzecha et al. 2010; Deboer 2011; Sibilia et al. 2012; Tsuchimochi et al. 2013; Theil et al. 2009).

When LPS was administered to healthy volunteers, it caused an early increase in ghrelin levels (Theil et al. 2009) (Maruna et al. 2005). Furthermore, neonates with infections and patients suffering from postoperative intra-abdominal sepsis have exhibited a significant rise in their ghrelin levels (Nematy et al. 2006; Arabi et al. 2019). Adult sepsis patients also displayed increased ghrelin levels, with an inverse correlation between ghrelin concentration, duration of ICU stay, and sepsis severity, determined by the Sequential Organ Failure Assessment (SOFA) score (Aziz et al. 2013). However, ICU patients had lower levels of ghrelin as compared to healthy controls (Nikitopoulou et al. 2020). Additionally, higher serum ghrelin levels at the time of hospital admission were found to be positively correlated with survival among sepsis patients. It was observed that patients with higher circulating ghrelin levels did not require mechanical ventilation when compared to those with lower levels. Moreover, there were no discernible differences in ghrelin concentrations between septic and non-septic critically ill patients (Seim et al. 2007).

## Renin angiotensin aldosterone (RAAS) axis

The Renin–Angiotensin–Aldosterone System (RAAS) is an important hormonal pathway responsible for maintaining blood pressure and fluid balance. Renin is released by the juxtaglomerular (JG) cells of nephrons in response to low blood pressure or volume. It cleaves angiotensinogen in the blood into angiotensin I (Ang I). Ang I is processed into angiotensin II (Ang II) by the angiotensin-convertase enzyme (ACE) found in the pulmonary endothelium. The RAAS axis is deranged during sepsis and is also involved in sepsis-induced acute kidney injury (S-AKI). Acute kidney injury (AKI) is a common complication of sepsis; about 57.7% of septic patients develop it (Uchino et al. 2005). An interplay of coagulative dysfunction, endothelial damage, and oxidative stress eventually causes tissue hypoxemia and leads to AKI during sepsis (Peerapornratana et al. 2019). In the early stages of sepsis, RAAS is activated in response to vasodilation, causing elevated levels of renin (Busse et al. 2023). Gleason et al. (2019) have shown that renin levels are a more reliable indicator of ICU mortality than lactate levels. Despite an elevation in renin levels, the response of the RAAS is insufficient to maintain blood pressure, which contributes to disease severity and increased mortality (Gleeson et al. 2019).

A recent literature review supports a proinflammatory role of Ang II associated with mortality and organ failure in sepsis as well (Jadhav and Sadaka 2019). Sepsis-induced vasodilation leads to overactivation of RAAS. Ang II upregulates the expression of tumor necrosis factor alpha (TNF-α) and interleukin-6 (IL-6), recruiting inflammatory cells to the kidney interstitium (Ruiz-Ortega et al. 2002). It also activates Nuclear Factor kappa β (NF-kβ) and intracellular adhesion molecules (ICAM) via angiotensin II type 2 receptor (AT2-R) and angiotensin II type 1 receptor (AT1-R), respectively, promoting pro-inflammatory gene expression and immune cell migration, thus furthering tissue damage and renal inflammation (Wolf et al. 2002; Pastore et al. 1999). Ang II also plays a direct role in the oxidative damage of renal cell components through ROS formation. These ROS influence the expression of inflammatory and extracellular matrix genes as well (Sachse and Wolf 2007).

Similarly, Ang II contributes to the formation of microthrombi and the overactivation of the coagulation cascade, mediated through the activation of AT1-R and upregulation of aldosterone. Al-Kadi et al., observed that administration of valsartan, an angiotensin receptor blocker (ARB), improved platelet adhesion, bleeding time, and levels of nitric oxide, thus reducing stasis-induced venous thrombotic changes (Gromotowicz-Poplawska et al. 2016). Ang II causes thrombus initiation and stabilization via AT2-R and angiotensinogen II type 4 receptor (AT4-R), respectively. AT2-R and AT4-R interact with bradykinin-1 and endothelin-1A receptors to mediate microvascular thrombus formation (Senchenkova et al. 2010). Moreover, aldosterone affects renal fibrosis via mineralocorticoid receptor (MR) activation. Aldosterone induces increased expression of inflammatory cytokines such as ICAM and TGF-beta and the generation of ROS (Martín-Fernandez et al. 2016).

Recent clinical trials showed that hospitalized septic patients given angiotensin-converting enzyme inhibitors (ACEIs) or ARBs are more likely to survive than patients not taking these drugs (Hsieh et al. 2020; Kim et al. 2019). A study in Female Wistar rats after cecal ligation and puncture (CLP) showed significant effects of different RAAS inhibitors; ramipril (ACEi), losartan (ARB), and spironolactone (aldosterone antagonist) on survival. (Al-Kadi et al. 2022) RAAS inhibitors reduce glomerular pressure and proteinuria and slow the progression of kidney fibrosis by limiting the effects of Ang II on efferent arteriole vasoconstriction and reducing the production of TGF-beta, which contributes to glomerular fibrosis (Zhang et al. 2017). However, RAAS inhibitors lower systemic blood pressure by antagonizing the effects of Ang II on vascular tone and aldosterone. This feature may exacerbate the profound hypotension seen during sepsis. Chronic use of ACEi/ARBs may put patients at risk for worse outcomes during sepsis due to RAAS inhibition. Alternatively, acute use of RAAS inhibitors during sepsis may not immediately affect blood pressure, and instead provide remediation for the oxidative, inflammatory, and coagulopathic effects of the RAAS (Lucas et al. 2006) (Fig. 4).

## Leptin

Leptin, derived from adipocytes, transmits peripheral metabolic information to the brain for the regulation of food intake, appetite and energy expenditure and participates in the neuroendocrine stress response (Mantzoros 1999; Flier 1998). Leptin is a stress-related hormone and affects the body’s response to injury in sepsis in addition to allowing fatty tissue stores in the periphery to communicate with the central nervous system (Tzanela et al. 2006).

**Fig. 5:**
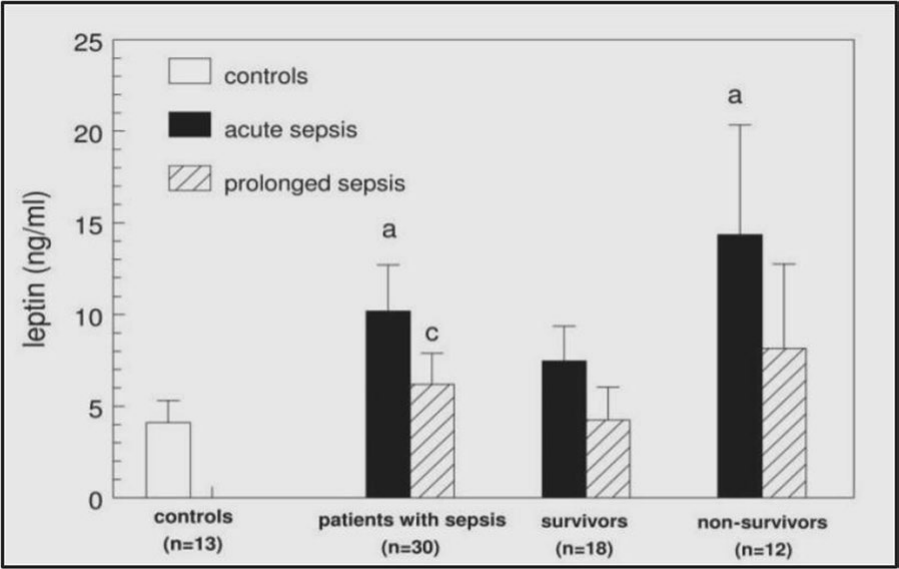
Leptin levels in control and different stages of sepsis (Acute and Prolonged phase) A: Statistically significant difference from controls, C: statistically significant difference from acute sepsis values. The leptin levels in patients with acute sepsis were higher than in controls (10.2 ± 2.5 vs. 4.1 ± 1.2 ng/ml, p = 0.01). Leptin in non-survivors was almost twofold higher than in survivors (14.35 ± 7.44 vs. 7.48 ± 1.9 ng/ml, but this difference did not reach statistical significance. A statistically significant decline in patient leptin levels was found during prolonged sepsis (from 10.2 ± 2.5 to 6.25 ± 1.7 ng/ml, p = 0.001). Controls (n = 13) Septic patients (n = 30) Survivors (n = 18) non-survivors (n = 12) Figure modified from Open access (IN VIVO journal) permissible to re-use under a CC-BY 4.0 license)

A study by Tzanela et al. measured leptin levels in acutely ill patients admitted to the ICU (= 30) and compared them with leptin levels in age- and gender- matched healthy control adults (n = 13). The acutely septic patients appeared to have increased levels of leptin compared to healthy controls, but the difference was not statistically significant. On the other hand, the acutely septic patients (within the 24 h of admission) had higher leptin levels than controls. Within 2 weeks, leptin levels decreased significantly. A graphical representation of these findings is shown in Fig. 5 (Tzanela et al. 2006; Arnalich et al. 1999). Arnalich et al. studied critically ill patients diagnosed with culture-proven bacteremia who met the diagnostic criteria for severe sepsis or septic shock, as defined by (American College of Chest Physicians/Society of Critical Care Medicine Consensus Conference, 1991, Northbrook, Illinois) the consensus conference. Plasma samples were collected within 48 h after blood culture collection, at the onset of systolic hypotension or septic shock, and then every 6 h (Bornstein et al. 1998). They demonstrated increased leptin levels during the acute phase and a decline in those patients who recovered within the 48-h study period. Borstein et al. also reported higher leptin levels in patients evaluated during acute sepsis (Papathanassoglou et al. 2001). A possible explanation for the biphasic response of leptin found in patients during the course of sepsis may be related to the increased release of inflammatory cytokines during the acute phase. These may cause the initial increase in leptin levels (Berghe 1998b). Later, other changes such as decreased activity of the GH axis and concomitant low IGF-I levels (Berghe 1998b) may mediate the decline in leptin levels. No correlation was demonstrated between circulating leptin and cortisol levels, either in the acute or in the prolonged septic phase, indicating that steroids do not participate in leptin alterations (Tzanela et al. 2006; Arnalich et al. 1999).

It was initially hypothesized that increased leptin levels during the acute phase exert protective effects, but no significant differences in leptin levels between survivors and non-survivors were observed, indicating potential confounding effects of other mediators. Multivariate analysis demonstrated that survival in septic patients was not related to absolute leptin levels or the decline of leptin during prolonged sepsis (Tzanela et al. 2006; Arnalich et al. 1999) (Fig. 5).

## Hypothalamus pituitary gonadal (HGA) axis

The hypothalamus secretes the gonadotropin-releasing hormone (GnRH), which then acts on the pituitary gland, promoting luteinizing hormone (LH) and follicle-stimulating hormone (FSH) secretion. In women, LH stimulates the ovary to produce androgens, which are then aromatized into estrogens under the control of FSH. In men, LH stimulates the Leydig cells in the testes to produce androgens. These androgens then act along with FSH to promote spermatogenesis. The androgens and estrogens inhibit the HPG axis through negative feedback (Spratt 2001; Guo et al. 1990).

In men, the cytokines released during acute sepsis promote peripheral aromatization, causing a decrease in testosterone concentration (Christeff et al. 1988). In septic patients, an increase in 17β-estradiol (E2) concentration and a simultaneous decrease in testosterone levels has been observed during septic shock (Berghe et al. 2001b). The peripheral aromatization continues into the chronic phase of sepsis, leading to extremely low levels of testosterone along with a drop in LH secretion (Dong et al. 1992). The decrease in testosterone is an adaptive response to sepsis since testosterone is an anabolic hormone. However, a long-term drop in testosterone can be detrimental. The mortality rates of men with sepsis have been found to be negatively correlated with testosterone concentrations (Mechanick and Nierman 2006).

In women, sepsis has been shown to decrease estrogen and progesterone concentrations. This is thought to be due to communication between the HPA and HPG axes in women (Breen and Karsch 2004). Corticotropin-releasing hormone (CRH) from the hypothalamus can inhibit GnRH release. In addition, the glucocorticoids released during sepsis inhibit LH secretion from the pituitary, eventually causing a drop in estrogen and progesterone release to some extent (Fleischmann et al. 2016).

A number of studies have documented an increase in morbidity and mortality rates in males vs females (Nasir et al. 2015; Xu et al. 2019; El-Lakany et al. 2018). Animal studies have shown a similar pattern. These are thought to be due to the cardioprotective effects of estrogen in females and the overall pro-inflammatory effects of estradiol, which promotes the phagocytic and bactericidal actions of immune cells (El-Lakany et al. 2018; Saia et al. 2015).

## Catecholamines

During the early phase of sepsis, catecholamines, either produced endogenously by the body or administered exogenously, are responsible for increasing heart rate and blood pressure to restore cardiovascular function. Because of their vasoactive properties, they also reduce blood flow to the skin and increase blood flow to the vital organs, such as the brain, heart, and kidneys, to maintain their perfusion in a state of shock (Backer and Foulon 2019; Rittirsch et al. 2008). Immunomodulatory effects of catecholamines are exerted through α- and β-adrenergic receptors expressed by immune cells. Stimulation of these receptors affects the functional responses of leukocytes, like cell proliferation and apoptosis. The release of pro-inflammatory cytokines like IL-1, IL-8 and TNF-α by macrophages and neutrophils is tightly regulated by α-adrenergic receptors, which in turn are regulated by catecholamines. In short, activation of the adrenergic pathways of the sympathetic nervous system in the early phase of sepsis promotes pro-inflammatory responses (Hartmann et al. 2017).

In early sepsis, at the cellular level, catecholamines increase metabolism by promoting hyperglycemia and hyperlactatemia. This increases oxygen demands when the body is already in a state of hypoperfusion, leading to further organ damage. Catecholamines aggravate mitochondrial uncoupling and contribute to oxidative stress, which leads to mitochondrial dysfunction (Hartmann et al. 2017). Catecholamines are affected by ROS resulting in the production of catecholamine-O-quinones and superoxide radicals that are thought to be responsible for catecholamine-induced cardiotoxicity through mitochondrial dysfunction. Alterations in Ca2 + handling and ROS lead to the opening of mitochondrial permeability transition pores (mPTPs), which results in the uncoupling of the mitochondria and eventually causing cell death. In early sepsis, catecholamines not only decrease splanchnic perfusion due to their vasoconstrictor properties but may also directly impair the motility of the gastrointestinal tract (Rudiger and Singer 2016).

Although necessary and lifesaving in the early flight or fight response to stressful stimuli, prolonged adrenergic activation through catecholamines may have deleterious effects and lead to organ dysfunction (Elenkov 2007). Endogenous catecholamines may selectively suppress cellular immunity and cause a shift toward the dominance of humoral immunity. They can boost regional immune responses through the induction of pro-inflammatory cytokines. Catecholamines tend to localize the inflammatory response through stimulation of more specific humoral immune responses while systemically suppressing Th1 responses, and in doing so, they protect the organism from the harmful effects of pro-inflammatory cytokines and other products of activated immune cells like macrophages (Lyte 1992). While catecholamines have both pro- and anti-inflammatory effects, current evidence strongly suggests immunosuppressive effects dominate in late sepsis, making patients vulnerable to secondary infections (Hahn et al. 1995).

Plasma catecholamine levels appear to be elevated in both early and late sepsis. Although the early hyperdynamic state may be mediated by catecholamines, no association seems to exist between the elevated catecholamine levels and the late hypodynamic state (Nishimura et al. 2000). In fact, a reduction in catecholamine concentration during late sepsis may lead to favorable results, but the short duration of one study makes it hard to trust the outcome (Elenkov 2007). More study needs to be done to have a better and complete understanding of the role of catecholamines during sepsis (Elenkov 2007).

## Fibroblast growth factor 21 (FGF21)

The Fibroblast Growth Factor (FGF) family consists of a number of metabolically active hormones with a variety of functions. The FGF21 was first discovered by Nishimura et al. (2000; Kharitonenkov et al. 2005) in mouse embryos and its possible role in metabolism was elucidated by studies conducted by Kharitonenkov et al. (Inagaki et al. 2007). Initial studies showed that FGF21 had many effects in rodent metabolism (especially diabetic and obese models) including decreasing blood glucose and triglycerides, inducing ketogenesis, gluconeogenesis, lipolysis, and lipid beta oxidation (Inagaki et al. 2008; Fazeli et al. 2015). In humans, FGF21 was hypothesized to act as a modulator of metabolic response to starvation because it was elevated after 7 days of fasting and enabled the body to utilize sources of fuel more effectively (Antonellis et al. 2016). Later studies in rodents have however discredited this idea showing that FGF21 knockout mice showed no change in their starvation response compared to normal mice (Patel et al. 2015). In humans, FGF21 is expressed in various organs including the liver, adipocytes, pancreas, blood brain barrier, skeletal muscle, testes, and heart (Hsuchou et al. 2007; Zhang et al. 2008; Johnson et al. 2009; Feingold et al. 2012).

A landmark study by Feingold et al. (Gariani et al. 2013) demonstrated that the levels of FGF21 doubled in rodents with sepsis induced by lipopolysaccharide, turpentine and zymosan, paving the way for a new treatment for sepsis and further research. The increase in FGF21 was mostly due to increase in production by skeletal muscles. The role of FGF21 in ketogenesis and starvation response was again brought into question because in FGF21 knockout mice, the ability of LPS to increase plasma free fatty acid levels was blunted. Treatment with exogenous FGF21 reduced the number of animals that die and the rapidity of death after LPS administration. FGF21 is also protected from the toxic effects of cecal ligation and puncture-induced sepsis. Thus, FGF21 is an acute phase reactant with positive effects that protects animals from the toxic effects of LPS and sepsis (Gariani et al. 2013).

Gariani et al. (Li et al. 2018) compared FGF21 levels in different groups of people admitted in the hospital. These included those in sepsis, those who fulfilled criteria for SIRS, and in healthy controls. They found that FGF21 levels were significantly higher in sepsis than in controls. FGF21 levels were higher in infectious than noninfectious sepsis. Interestingly, FGF21 levels correlated with APACHE-2 score, but not with procalcitonin and CRP which are traditional markers of inflammation. This indicates that FGF21 may be an early marker of sepsis, showing raised levels even before other markers do (Li et al. 2018).

Later studies tried to determine whether the absolute value of FGF21 (Li et al. 2021) and a large, acute increase in FGF21 levels (14) could predict 28-day mortality risk in patients admitted to the ICU with sepsis. ROC curves for FGF21 and 28-day mortality had high sensitivity and specificity. Patients with FGF21 values < 3200 pg/ml had significantly better outcomes than those with a value over this cutoff, indicating that FGF21 was an independent predictor of 28-day mortality. Similarly, in patients with Sepsis and ARDS, non-survivors had a FGF21 value four times greater than in the survivors. The serum values of FGF21 decreased progressively in the survivors while the non-survivors showed a progressively rising FGF21 level (Li et al. 2021).

Newer roles of FGF21 are emerging in critical diseases including acute lung injury/acute respiratory distress syndrome (ALI/ARDS), acute myocardial injury (AMI), acute kidney injury (AKI), sepsis, and liver failure (Yan et al. 2022). This seems like an exciting arena of research that may result in drastic improvements in the management of these serious conditions (Yan et al. 2022).

## Protein catabolism

One of the main characteristics of the metabolic reaction to sepsis is skeletal muscle protein catabolism. When an endotoxin or an intra-abdominal abscess induces sepsis, translational efficiency is reduced inhibiting muscle protein synthesis. The data suggests that sepsis reduces the rate of protein synthesis in fast-twitch muscles by inhibiting peptide-chain initiation relative to elongation (Jepson et al. 1986; Vary and Kimball 1992; Jurasinski et al. 1995; Vary et al. 1996).

Sepsis seems to exhibit a preference for enhancing the degradation of myofibrillar proteins. Escalation of myofibrillar protein degradation (200–400%) in the extensor digitorum longus (EDL) significantly surpasses the rise in total protein breakdown (50%) observed during sepsis initiation through cecal ligation and puncture (Vary et al. 1996; Hasselgren et al. 1986, 1989; Tiao et al. 1994) or subsequent to sepsis induction via lipopolysaccharide injection (Zamir et al. 1992). Skeletal muscle possesses various proteolytic mechanisms that can degrade cellular proteins such as lysosomal (Zamir et al. 1992; Bird et al. 1980; Ruff and Secrist 1984; Hummel 1988), calcium-dependent cysteine proteinases, and calpains (Sorimachi and Suzuki 1992; Benson et al. 1989; Bhattacharyya et al. 1991) or ROS (Gecha and Fagan 1992).

Glucocorticoids play a significant role in the regulation of both protein synthesis and protein degradation within skeletal muscle. The increased levels of glucocorticoids in the bloodstream are considered an initial response to invasive triggers in patients with trauma or sepsis. When steroids are administered to either healthy human subjects or rats, there is a notable increase in the breakdown of muscle protein, achieved through the dual mechanism of inhibiting protein synthesis and promoting degradation. Similar to the effects observed in cases of sepsis, the breakdown of myofibrillar proteins is heightened following the administration of glucocorticoids (Darmaun et al. 1988; Rannels and Jefferson 1980; Kelly and Goldspink 1982; Kayali et al. 1987).

The proinflammatory cytokines, including TNF, IL-1, and IL-6, have been identified as potential mediators of the metabolic disturbances in sepsis. Clowes and colleagues illustrated that exposure of muscle tissue from healthy subjects to plasma obtained from septic individuals in a controlled environment led to acceleration in protein breakdown. Cytokines, particularly TNF and IL-1, play a significant role in the findings reported by Clowes et al. (1983). The introduction of a tumor that secretes TNF results in notable muscle wasting in mice (Tracey et al. 1990), and administration of TNF or IL-1 in living organisms leads to an increase in nitrogen excretion from skeletal muscle and a decline in body protein levels in otherwise unaffected animals (Fong et al. 1989; Hoshino et al. 1991).

## Lipid metabolism

In acute sepsis, lipid breakdown is increased. Although the exact mechanism is unknown, three different pathophysiological changes in the body may be responsible for this. First, the activation of hormone-sensitive lipase (HSL) through the lipopolysaccharide (LPS) component of microbes promotes lipolysis (Wasyluk and Zwolak 2021; Muniz-Santos et al. 2023). Second, there is an increase in stress hormones, including cortisol, growth hormone, glucagon, and catecholamines, during sepsis, which themselves have lipolytic properties (Wasyluk and Zwolak 2021). Third, insulin resistance during sepsis blunts the lipogenic activity of insulin and promotes lipolysis (Wasyluk and Zwolak 2021; Muniz-Santos et al. 2023). Despite increased lipolysis, however, β-oxidation of fatty acids is downregulated. This is due to disruption of the peroxisome proliferator-activated α receptor (PPAR-α), which is the main transcription factor involved in regulating β-oxidation (Wasyluk and Zwolak 2021).

Levels of lipoproteins are variably affected in sepsis. Concentration of very low-density lipoprotein (VLDL) is increased. On the other hand, high (HDL) and low-density (LDL) lipoprotein levels, which themselves have the ability to antagonize lipopolysaccharide (LPS) and have anti-inflammatory properties, are decreased (Wasyluk and Zwolak 2021). In fact, levels of HDL can be used as prognostic markers for mortality and multi-organ dysfunction during sepsis (Muniz-Santos et al. 2023). Levels of total plasma cholesterol also decrease in inverse proportion to the severity of sepsis and can be used as a predictive factor in the prognosis of acute sepsis (Hofmaenner et al. 2021).

Sepsis is associated with the accumulation of lipids in multiple organs throughout the body. For example, the livers of patients with sepsis have been shown to have an excess buildup of fat (Koskinas et al. 2008). Similarly, cardiac myocytes shift their usual source of energy from fatty acid oxidation in the healthy state to glycolysis during sepsis, resulting in the accumulation of free fatty acids (FFA) in the heart (Rossi et al. 2007). Experiments performed in the male CD-1 mice have shown excess triglycerides in the renal cortex after injecting them with lipopolysaccharides (LPS) (Richard et al. 2005). Lipid accumulation outside adipose tissue is associated with lipotoxicity, mitochondrial dysfunction, and lipoapoptosis (Wasyluk and Zwolak 2021).

## Acid–base disturbances

In intensive care units, patients with sepsis or acute inflammatory conditions commonly have disturbances in acid base balance. Maintaining this balance can be difficult. There are different types of acid–base imbalances, including metabolic acidosis, metabolic alkalosis, respiratory acidosis, respiratory alkalosis, and mixed acid base disturbances. High anion gap metabolic acidosis is the most common disturbance in severely ill septic patients. It may result in acidosis from lactic acid buildup inside the cell and insufficient oxygen circulation generates hypoxia in tissues and anaerobic glycolysis in the cells, causing hyperlactatemia, lactic acidosis and serum pH < 7.35. or hyperchloremic non-anion gap metabolic acidosis which is due to fluid resuscitation following chloride rich solutions and complicates patient management. (chloride-associated acidosis) (Ganesh et al. 2016; Noritomi 2009). In sepsis, lactic acid levels are used as a prognostic or diagnostic indicator. A decrease in serum lactate acid levels and an increase in bicarbonate (HCO3) levels over the course of 5–6 days are considered effective prognostic indicators for survival and good outcomes in septic patients (Achanti and Szerlip 2022; Rababa et al. 2022).

## Current management and role of insulin

Sepsis is a life-threatening medical condition that is associated with numerous complications, including multiorgan failure and septic shock (Rababa et al. 2022). Early therapeutic and diagnostic interventions are crucial to reducing mortality and morbidity in patients. Fluid resuscitation and antibiotic administration are the cornerstones of the management of early phase sepsis (Rhodes et al. 2017; Evans et al. 2021; Shapiro et al. 2023; Meyer and Prescott 2024).

## Endocrine manipulations: insulin therapy in sepsis

In patients with sepsis, peripheral insulin resistance mediated by a high amount of proinflammatory cytokines is a well-known cause of dysglycemia. This necessitates the need for optimal control of blood glucose levels for better patient outcomes. Earlier studies have established that intensive insulin therapy has no significant benefits in critically ill patients, whether septic or not, and poses a greater risk for hypoglycemia. This was further endorsed in a recent meta-analysis by Meng et al. who showed that intensive and liberal blood glucose control had comparable effects on improving outcomes in septic patients, with a greater risk of hypoglycemia in the former. The case fatality due to severe hypoglycemia in both groups was similar however in the overall population, the mortality rate of severe hypoglycemia was higher in the patients with intensive glycemic control, primarily due to high incidence of hypoglycemia in this group (Meng et al. 2024). The FORECAST study evaluated the incidence of dysglycemia after admission and the impact of hyperglycemia as well as hypoglycemia on outcomes in nondiabetic and diabetic patients with sepsis. The study showed that in nondiabetic patients, early hypoglycemic episodes and late hyperglycemia were positively associated with mortality, and no such correlation was observed in diabetic individuals. (Abe et al. 2018) and showed that, among non-diabetic individuals hypoglycemia and sepsis combined posed a 2.5 times higher risk of mortality than those without hypoglycemia and sepsis (Abe et al. 2018). The 2021 SSC guidelines recommend a glucose level of 144–180 mg/dl in septic patients. Current guidelines suggest the start of insulin therapy when there is persistent hyperglycemia (> 180 mg/dl) and avoidance of hypoglycemia (< 80 mg/dl). Intensive glycemic control (110–140 mg/dl) can be tried in a selective group of critically ill patients without avoiding significant hypoglycemia (Song et al. 2014; Berghe et al. 2003, 2006; Finfer et al. 2009; Evans et al. 2021; Marik and Raghavan 2004; Dandona et al. 2001; Brunkhorst et al. 2008; Gunst et al. 2023).

## Conclusions

Severe sepsis and septic shock trigger endocrine changes. These can be so extreme that they exceed the capacity of endocrine organs to make and release hormones. This may result in hypercortisolemia, euthyroid sick syndrome, insulin resistance, hyperglycemia, hyperleptinemia, abnormally low vasopressin levels and several other conditions. There are few generally accepted treatments for these problems despite the fact that they negatively impact the host and intensify the multi-organ failure seen in sepsis. A clinical assessment of sepsis can be challenging because of patient-to-patient differences in hormone pharmacology, drug/drug interactions, circadian rhythms, and laboratory test results may complicate management. Endocrine biomarkers during sepsis therapy may help one assess disease severity.

## Data Availability

No datasets were generated or analysed during the current study.

## Abbreviations

AR: Adrenergic receptor
ATP: Adenosine triphosphate
BAT: Brown adipose tissue
DC: Dendritic cell
ETC: Electron transport chain
NK: Natural killer cell
ROS: Reactive oxygen species
Th: T-helper cell
Treg: Regulatory T-cell
UCP: Uncoupling protein
SSC: Surviving sepsis campaign
CLP: Cecal ligation and puncture
EGDT: Early Goal-Directed Therapy
HPA: Hypothalamic–pituitary–adrenal axis
HPT: Hypothalamic-Pituitary-Thyroid Axis
GH: Growth hormone
IGF-1: Insulin-like growth factor-1
RAAS: Renin–angiotensin–aldosterone system
ACTH: Adrenocorticotropic hormone
CRH: Corticotropin-releasing hormone
CBG: Cortisol-binding globulin
TRH: Thyrotropin-releasing hormone
TSH: Thyroid-stimulating hormone
T3: Triiodothyronine
T4: Thyroxine
rT3: Reverse triiodothyronine
TNF-α: Tumor necrosis factor-alpha
IL-5: Interleukin-5
IL-6: Interleukin-6
NF-κB: Nuclear factor kappa B
ICAM: Intracellular adhesion molecule
PI3K: Phosphatidylinositide 3-Kinase
mTOR: Mammalian target of rapamycin
cAMP: Cyclic adenosine monophosphate
PKA: Protein kinase A
CaMKK: Calcium/calmodulin-dependent protein kinase kinase
NRF2: Nuclear Factor Erythroid 2-Related Factor 2
G6PD: Glucose-6-Phosphate Dehydrogenase
2-DG: 2-Deoxy-D-Glucose
MAVS: Mitochondrial antiviral signaling
HK: Hexokinase
RIG-I: Retinoic Acid-Inducing Gene I
HIF-1α: Hypoxia-inducible factor-1 alpha
mTORC1&2: Mammalian target of rapamycin complex 1 and 2
GP73: Golgi Protein 73
GLUT4: Glucose Transporter Type 4
AKI: Acute kidney injury
AT1-R: Angiotensin II type 1 receptor
AT2-R: Angiotensin II type 2 receptor
AT4-R: Angiotensin II type 4 receptor
ARB: Angiotensin receptor blocker
MR: Mineralocorticoid receptor
TGF-β: Transforming growth factor beta
ROS: Reactive oxygen species
PEEP: Positive End-Expiratory Pressure
VTE: Venous Thromboembolism
HDL: High-density lipoprotein
LDL: Low-density lipoprotein
VLDL: Very low-density lipoprotein
LPS: Lipopolysaccharide
FFA: Free fatty acids
RIG-1: (RIG-I (Retinoic Acid-inducible Gene-I)

## References

[CR1] Abe T, Ogura H, Shiraishi A, et al. Characteristics, management, and in-hospital mortality among patients with severe sepsis in intensive care units in Japan: the FORECAST study. Crit Care. 2018;22(1):322. 10.1186/s13054-018-2186-7. 30466493 10.1186/s13054-018-2186-7 PMC6251147

[CR2] Achanti AT, Szerlip HM. Acid-base disorders in the critically Ill patient. Clin J Am Soc Nephrol. 2022. 10.2215/CJN.04500422. 35998977 10.2215/CJN.04500422 PMC10101555

[CR3] Agwunobi AO, Reid C, Maycock P, et al. Insulin resistance and substrate utilization in human endotoxemia. J Clin Endocrinol Metab. 2000;85(10):3770–8. 11061537 10.1210/jcem.85.10.6914

[CR4] Alberti C, Brun-Buisson C, Burchardi H, et al. Erratum to: epidemiology of sepsis and infection in ICU patients from an international multicentre cohort study. Intensive Care Med. 2002;28(4):525–6. 10.1007/s00134-002-1284-8. 10.1007/s00134-001-1143-z 11907653

[CR5] Al-Kadi A, El-Daly M, El-Tahawy NFG, Khalifa MMA, Ahmed AF. Angiotensin aldosterone inhibitors improve survival and ameliorate kidney injury induced by sepsis through suppression of inflammation and apoptosis. Fundam Clin Pharmacol. 2022;36(2):286–95. 10.1111/fcp.12718. 34309069 10.1111/fcp.12718

[CR6] American Diabetes Association. Classification and diagnosis of diabetes: standards of medical care in diabetes-2020. Diabetes Care. 2020;43(Suppl 1):S14–31. 10.2337/dc20-S002. 31862745 10.2337/dc20-S002

[CR7] Amunugama K, Pike DP, Ford DA. The lipid biology of sepsis. J Lipid Res. 2021;62:100090. 34087197 10.1016/j.jlr.2021.100090 PMC8243525

[CR8] Andreis DT, Singer M. Catecholamines for inflammatory shock: a Jekyll-and-Hyde conundrum. Intensive Care Med. 2016;42(9):1387–97. 10.1007/s00134-016-4249-z. 26873833 10.1007/s00134-016-4249-z

[CR9] Angus DC, van der Poll T. Severe sepsis and septic shock. N Engl J Med. 2013;369(9):840–51. 10.1056/NEJMra1208623. 23984731 10.1056/NEJMra1208623

[CR10] Angus DC, Linde-Zwirble WT, Lidicker J, et al. Epidemiology of severe sepsis in the United States: analysis of incidence, outcome, and associated costs of care. Crit Care Med. 2001;29:13031310. 10.1097/00003246-200107000-00002 11445675

[CR11] Annane D, Sébille V, Charpentier C, et al. Effect of treatment with low doses of hydrocortisone and fludrocortisone on mortality in patients with septic shock. JAMA. 2002;288(7):862–71. 10.1001/jama.288.7.862. 12186604 10.1001/jama.288.7.862

[CR12] Annane D, Pastores SM, Arlt W, et al. Critical illness-related corticosteroid insufficiency (CIRCI): a narrative review from a multispecialty task force of the society of critical care medicine (SCCM) and the European society of intensive care medicine (ESICM). Intensive Care Med. 2017;43(12):1781–92. 10.1007/s00134-017-4914-x. 28940017 10.1007/s00134-017-4914-x

[CR13] Antonellis PJ, Hayes MP, Adams AC. fibroblast growth factor 21-null mice do not exhibit an impaired response to fasting. Front Endocrinol. 2016;7:77. 10.3389/fendo.2016.00077. 10.3389/fendo.2016.00077 PMC4928592 27445980

[CR14] Arabi YM, Jawdat D, Al-Dorzi HM, et al. Leptin, ghrelin, and leptin/ghrelin ratio in critically Ill patients. Nutrients. 2019;12(1):36. 10.3390/nu12010036. 31877773 10.3390/nu12010036 PMC7020071

[CR15] Arafah BM. Hypothalamic pituitary adrenal function during critical illness: limitations of current assessment methods. J Clin Endocrinol Metab. 2006;91(10):3725–45. 10.1210/jc.2006-0674. 16882746 10.1210/jc.2006-0674

[CR16] Arnalich F, López J, Codoceo R, Jiménez M, Madero R, Montiel C. Relationship of plasma leptin to plasma cytokines and human survival in sepsis and septic shock. J Infect Dis. 1999;180(3):908–11. 10.1086/314963. 10438392 10.1086/314963

[CR17] Aziz M, Jacob A, Yang WL, Matsuda A, Wang P. Current trends in inflammatory and immunomodulatory mediators in sepsis. J Leukoc Biol. 2013;93(3):329–42. 10.1189/jlb.0912437. 23136259 10.1189/jlb.0912437 PMC3579020

[CR18] Bauza-Martinez J, Aletti F, Pinto BB, et al. Proteolysis in septic shock patients: plasma peptidomic patterns are associated with mortality. Br J Anaesth. 2018;121(5):1065–74. 30336851 10.1016/j.bja.2018.05.072

[CR19] Benson DW, Hasselgren PO, Hiyama DT, James JH, Li S, Rigel DF, Fischer JE. Effect of sepsis on calcium uptake and content in skeletal muscle and regulation in vitro by calcium of total and myofibrillar protein breakdown in control and septic muscle: results from a preliminary study. Surgery. 1989;106(1):87–93. 2740990

[CR20] Bentham J, Rodriguez-Arnao J, Ross RJ. Acquired growth hormone resistance in patients with hypercatabolism. Horm Res. 1993;40(1–3):87–91. 10.1159/000183772. 7507879 10.1159/000183772

[CR21] Berneis K, Keller U. Metabolic actions of growth hormone: direct and indirect. Baillieres Clin Endocrinol Metab. 1996;10(3):337–52. 10.1016/s0950-351x(96)80470-8. 8853443 10.1016/s0950-351x(96)80470-8

[CR22] Bessey PQ, Watters JM, Aoki TT, Wilmore DW. Combined hormonal infusion simulates the metabolic response to injury. Ann Surg. 1984;200(3):264–81. 10.1097/00000658-198409000-00004. 6431917 10.1097/00000658-198409000-00004 PMC1250469

[CR23] Bhattacharyya J, Thompson K, Sayeed MM. Calcium-dependent and calcium-independent protease activities in skeletal muscle during sepsis. Circ Shock. 1991;35(2):117–22. 1777946

[CR24] Bird JW, Carter JH, Triemer RE, Brooks RM, Spanier AM. Proteinases in cardiac and skeletal muscle. Fed Proc. 1980;39(1):20–5. 6985869

[CR25] Bone RC. Gram-negative sepsis: a dilemma of modern medicine. Clin Microbiol Rev. 1993;6(1):57–68. 10.1128/CMR.6.1.57. 8457980 10.1128/cmr.6.1.57 PMC358266

[CR26] Boonen E, Van den Berghe G. Mechanisms in endocrinology: new concepts to further unravel adrenal insufficiency during critical illness. Eur J Endocrinol. 2016;175(1):R1–9. 10.1530/EJE-15-1098. 26811405 10.1530/EJE-15-1098

[CR27] Boonen E, Vervenne H, Meersseman P, et al. Reduced cortisol metabolism during critical illness. N Engl J Med. 2013;368(16):1477–88. 10.1056/NEJMoa1214969. 23506003 10.1056/NEJMoa1214969 PMC4413428

[CR28] Boonen E, Meersseman P, Vervenne H, Meyfroidt G, Guïza F, Wouters PJ, Veldhuis JD, Van den Berghe G. Reduced nocturnal ACTH-driven cortisol secretion during critical illness. Am J Physiol Endocrinol Metab. 2014;306(8):E883–92. 10.1152/ajpendo.00009.2014. 24569590 10.1152/ajpendo.00009.2014 PMC3989736

[CR29] Bornstein SR, Licinio J, Tauchnitz R, et al. Plasma leptin levels are increased in survivors of acute sepsis: associated loss of diurnal rhythm in cortisol and leptin secretion. J Clin Endocrinol Metab. 1998;83(1):280–3. 10.1210/jcem.83.1.4610. 9435456 10.1210/jcem.83.1.4610

[CR30] Breen KM, Karsch FJ. Does cortisol inhibit pulsatile luteinizing hormone secretion at the hypothalamic or pituitary level? Endocrinology. 2004;145(2):692–8. 10.1210/en.2003-1114. 14576178 10.1210/en.2003-1114

[CR31] Brunkhorst FM, Engel C, Bloos F, Meier-Hellmann A, Ragaller M, Weiler N, et al. Intensive insulin therapy and pentastarch resuscitation in severe sepsis. N Engl J Med. 2008;358(2):125–39. 18184958 10.1056/NEJMoa070716

[CR32] Busse LW, Schaich CL, Chappell MC, McCurdy MT, Staples EM, Teri Lohuis CD, Hinson JK, Severansky JE, Rothman RE, Wright DW, Marin GS, Khanna AK. Association, of active renin content with mortality in critically ill patients: a post-hoc analysis of the VICTAS trial. Crit Care Med. 2023;50:1–11. 10.1097/CCM.0000000000006095 PMC10876175 37947484

[CR33] Ceriello A, Genovese S, Mannucci E, Gronda E. Glucagon and heart in type 2 diabetes: new perspectives. Cardiovasc Diabetol. 2016;15(1):123. 10.1186/s12933-016-0440-3. 27568179 10.1186/s12933-016-0440-3 PMC5002329

[CR34] Chang YW, Hung LC, Chen YC, et al. Insulin reduces inflammation by regulating the activation of the NLRP3 inflammasome. Front Immunol. 2021;11:587229. 10.3389/fimmu.2020.587229. 33679687 10.3389/fimmu.2020.587229 PMC7933514

[CR35] Chen GY, Núñez G. Sterile inflammation: sensing and reacting to damage. Nat Rev Immunol. 2010;10(12):826–37. 10.1038/nri2873. 21088683 10.1038/nri2873 PMC3114424

[CR36] Chen Y, Huang L, Qi X, et al. Insulin receptor trafficking: consequences for insulin sensitivity and diabetes. Int J Mol Sci. 2019;20(20):5007. 31658625 10.3390/ijms20205007 PMC6834171

[CR37] Chorny A, Anderson P, Gonzalez-Rey E, Delgado M. Ghrelin protects against experimental sepsis by inhibiting high-mobility group box 1 release and by killing bacteria. J Immunol. 2008;180(12):8369–77. 10.4049/jimmunol.180.12.8369. 18523304 10.4049/jimmunol.180.12.8369

[CR38] Christeff N, Benassayag C, Carli-Vielle C, Carli A, Nunez EA. Elevated oestrogen and reduced testosterone levels in the serum of male septic shock patients. J Steroid Biochem. 1988;29(4):435–40. 10.1016/0022-4731(88)90254-3. 3374133 10.1016/0022-4731(88)90254-3

[CR39] Clowes GH Jr, George BC, Villee CA Jr, Saravis CA. Muscle proteolysis induced by a circulating peptide in patients with sepsis or trauma. N Engl J Med. 1983;308(10):545–52. 10.1056/NEJM198303103081001. 6828080 10.1056/NEJM198303103081001

[CR40] Cooper MS, Stewart PM. Corticosteroid insufficiency in acutely ill patients. N Engl J Med. 2003;348(8):727–34. 10.1056/NEJMra020529. 12594318 10.1056/NEJMra020529

[CR41] Costantini E, Carlin M, Porta M, Brizzi MF. Type 2 diabetes mellitus and sepsis: state of the art, certainties and missing evidence. Acta Diabetol. 2021;58(9):1139–51. 10.1007/s00592-021-01728-4. 33973089 10.1007/s00592-021-01728-4 PMC8316173

[CR42] Dandona P, Aljada A, Mohanty P, Ghanim H, Hamouda W, Assian E, et al. Insulin inhibits intranuclear nuclear factor kappaB and stimulates IkappaB in mononuclear cells in obese subjects: evidence for an anti-inflammatory effect? J Clin Endocrinol Metab. 2001;86(7):3257–65. 11443198 10.1210/jcem.86.7.7623

[CR43] Darmaun D, Matthews DE, Bier DM. Physiological hypercortisolemia increases proteolysis, glutamine, and alanine production. Am J Physiol. 1988;255(3 Pt 1):E366–73. 10.1152/ajpendo.1988.255.3.E366. 3048115 10.1152/ajpendo.1988.255.3.E366

[CR44] Date Y, Kojima M, Hosoda H, et al. Ghrelin, a novel growth hormone-releasing acylated peptide, is synthesized in a distinct endocrine cell type in the gastrointestinal tracts of rats and humans. Endocrinology. 2000;141(11):4255–61. 10.1210/endo.141.11.7757. 11089560 10.1210/endo.141.11.7757

[CR45] De Costa CM. The contagiousness of childbed fever: a short history of puerperal sepsis and its treatment. Med J Aust. 2002;177(11–12):668–71. 10.5694/j.1326-5377.2002.tb05004.x. 12463995 10.5694/j.1326-5377.2002.tb05004.x

[CR46] De Backer D, Foulon P. Minimizing catecholamines and optimizing perfusion. Crit Care. 2019;23(Suppl1):149. 31200777 10.1186/s13054-019-2433-6 PMC6570631

[CR47] de Groof F, Joosten KF, Janssen JA, et al. Acute stress response in children with meningococcal sepsis: important differences in the growth hormone/insulin-like growth factor I axis between nonsurvivors and survivors. J Clin Endocrinol Metab. 2002;87(7):3118–24. 10.1210/jcem.87.7.8605. 12107211 10.1210/jcem.87.7.8605

[CR48] Deboer MD. Use of ghrelin as a treatment for inflammatory bowel disease: mechanistic considerations. Int J Pept. 2011;2011:189242. 10.1155/2011/189242. 21845198 10.1155/2011/189242 PMC3154487

[CR49] Delile E, Neviere R, Thiebaut PA, et al. Reduced insulin resistance contributes to the beneficial effect of protein tyrosine phosphatase-1b deletion in a mouse model of sepsis. Shock. 2017;48(3):355–63. 28272165 10.1097/SHK.0000000000000853

[CR50] Delporte C. Structure and physiological actions of ghrelin. Scientifica. 2013;2013:518909. 10.1155/2013/518909. 24381790 10.1155/2013/518909 PMC3863518

[CR51] den Brinker M, Joosten KF, Visser TJ, et al. Euthyroid sick syndrome in meningococcal sepsis: the impact of peripheral thyroid hormone metabolism and binding proteins. J Clin Endocrinol Metab. 2005;90(10):5613–20. 10.1210/jc.2005-0888. 16076941 10.1210/jc.2005-0888

[CR52] Desborough JP. The stress response to trauma and surgery. Br J Anaesth. 2000;85(1):109–17. 10.1093/bja/85.1.109. 10927999 10.1093/bja/85.1.109

[CR53] Dong Q, Hawker F, McWilliam D, Bangah M, Burger H, Handelsman DJ. Circulating immunoreactive inhibin and testosterone levels in men with critical illness. Clin Endocrinol. 1992;36(4):399–404. 10.1111/j.1365-2265.1992.tb01466.x. 10.1111/j.1365-2265.1992.tb01466.x 1424172

[CR54] Elenkov IJ. Effects of catecholamines on the immune response. In: NeuroImmune biology, vol. 7. Amsterdam: Elsevier; 2007. p. 189–206.

[CR55] El-Lakany MA, Fouda MA, El-Gowelli HM, El-Gowilly SM, El-Mas MM. Gonadal hormone receptors underlie the resistance of female rats to inflammatory and cardiovascular complications of endotoxemia. Eur J Pharmacol. 2018;823:41–8. 10.1016/j.ejphar.2018.01.051. 29382531 10.1016/j.ejphar.2018.01.051

[CR56] Ellger B, Westphal M, Stubbe HD, Van den Heuvel I, Van Aken H, Van den Berghe G. Glycemic control in sepsis and septic shock: friend or foe? Anaesthesist. 2008;57(1):43–8. 18034219 10.1007/s00101-007-1285-7

[CR57] Evans L, Rhodes A, Alhazzani W, et al. Surviving sepsis campaign: international guidelines for management of sepsis and septic shock 2021. Intensive Care Med. 2021;47(11):1181–247. 10.1007/s00134-021-06506-y. 34599691 10.1007/s00134-021-06506-y PMC8486643

[CR58] Fazeli PK, Lun M, Kim SM, Bredella MA, Wright S, Zhang Y, et al. FGF21 and the late adaptive response to starvation in humans. J Clin Invest. 2015;125:4601–11. 10.1172/JCI83349. 26529252 10.1172/JCI83349 PMC4665770

[CR59] Feingold KR, Grunfeld C, Heuer JG, Gupta A, Cramer M, Zhang T, Shigenaga JK, Patzek SM, Chan ZW, Moser A, Bina H, Kharitonenkov A. FGF21 is increased by inflammatory stimuli and protects leptin-deficient ob/ob mice from the toxicity of sepsis. Endocrinology. 2012;153(6):2689–700. 10.1210/en.2011-1496. 22474187 10.1210/en.2011-1496 PMC3359613

[CR60] Ferreira BL, Sousa MB, Leite GGF, Brunialti MKC, Nishiduka ES, Tashima AK, van der Poll T, Salomão R. Glucose metabolism is upregulated in the mononuclear cell proteome during sepsis and supports endotoxin-tolerant cell function. Front Immunol. 2022;13:1051514. 10.3389/fimmu.2022.1051514. 36466921 10.3389/fimmu.2022.1051514 PMC9718365

[CR61] Fleischmann C, Thomas-Rueddel DO, Hartmann M, et al. Hospital incidence and mortality rates of sepsis. Dtsch Arztebl Int. 2016;113(10):159–66. 10.3238/arztebl.2016.0159. 27010950 10.3238/arztebl.2016.0159 PMC4814768

[CR62] Flier JS. What’s in a name? in search of Leptin’s physiologic role. J Clin Endocrinol Metab. 1998;83(5):1407–13. 10.1210/jc.83.5.1407. 9589630 10.1210/jcem.83.5.4779

[CR63] Fliers E, Guldenaar SE, Wiersinga WM, Swaab DF. Decreased hypothalamic thyrotropin-releasing hormone gene expression in patients with nonthyroidal illness. J Clin Endocrinol Metab. 1997;82(12):4032–6. 10.1210/jcem.82.12.4404. 9398708 10.1210/jcem.82.12.4404

[CR64] Foks M, Dudek A, Polok K, Nowak-Kózka I, Fronczek J, Szczeklik W. Thyroid hormones as potential prognostic factors in sepsis. Anaesthesiol Intensive Ther. 2019;51(3):205–9. 10.5114/ait.2019.86883. 31418258 10.5114/ait.2019.86883

[CR65] Fong Y, Moldawer LL, Marano M, Wei H, Barber A, Manogue K, Tracey KJ, Kuo G, Fischman DA, Cerami A, et al. Cachectin/TNF or IL-1 alpha induces cachexia with redistribution of body proteins. Am J Physiol. 1989;256(3 Pt 2):R659–65. 10.1152/ajpregu.1989.256.3.R659. 2784290 10.1152/ajpregu.1989.256.3.R659

[CR66] Funk DJ, Parrillo JE, Kumar A. Sepsis and septic shock: a history. Crit Care Clin. 2009;25(1):83–101. 10.1016/j.ccc.2008.12.003. 19268796 10.1016/j.ccc.2008.12.003

[CR67] Galley HF. Oxidative stress and mitochondrial dysfunction in sepsis. Br J Anaesth. 2011;107(1):57–64. 10.1093/bja/aer093. 21596843 10.1093/bja/aer093

[CR68] Galsgaard KD, Pedersen J, Knop FK, Holst JJ, Wewer Albrechtsen NJ. Glucagon receptor signaling and lipid metabolism. Front Physiol. 2019;10:413. 10.3389/fphys.2019.00413. 31068828 10.3389/fphys.2019.00413 PMC6491692

[CR69] Ganesh K, Sharma RN, Pillai MG. A profile of metabolic acidosis in patients with sepsis in an intensive care unit setting. Int J Crit Illness Injury Sci. 2016. 10.4103/2229-5151.195417. 10.4103/2229-5151.195417 PMC5225760 28149822

[CR70] Gariani K, Drifte G, Dunn-Siegrist I, Pugin J, Jornayvaz F. Increased FGF21 plasma levels in humans with sepsis and SIRS. Endocr Connect. 2013;2:146–53. 10.1530/EC-13-0040. 23999826 10.1530/EC-13-0040 PMC3845842

[CR71] Gecha OM, Fagan JM. Protective effect of ascorbic acid on the breakdown of proteins exposed to hydrogen peroxide in chicken skeletal muscle. J Nutr. 1992;122(11):2087–93. 10.1093/jn/122.11.2087. 1432249 10.1093/jn/122.11.2087

[CR72] Gerich JE, Lorenzi M, Hane S, Gustafson G, Guillemin R, Forsham PH. Evidence for a physiologic role of pancreatic glucagon in human glucose homeostasis: studies with somatostatin. Metabolism. 1975;24(2):175–82. 10.1016/0026-0495(75)90018-9. 1113681 10.1016/0026-0495(75)90018-9

[CR73] Gheorghiţă V, Barbu AE, Gheorghiu ML, Căruntu FA. Endocrine dysfunction in sepsis: a beneficial or deleterious host response? Germs. 2015;5(1):17–25. 10.11599/germs.2015.1067. 25763364 10.11599/germs.2015.1067 PMC4350863

[CR74] Giustina A, Veldhuis JD. Pathophysiology of the neuroregulation of growth hormone secretion in experimental animals and the human. Endocr Rev. 1998;19(6):717–97. 10.1210/edrv.19.6.0353. 9861545 10.1210/edrv.19.6.0353

[CR75] Gleeson PJ, Crippa IA, Mongkolpun W, Cavicchi FZ, Van Meerhaeghe T, Brimioulle S, Taccone FS, Vincent JL, Creteur J. Renin as a marker of tissue perfusion and prognosis in critically ill patients. Crit Care Med. 2019;47(2):152–8. 30653055 10.1097/CCM.0000000000003544

[CR76] Granado M, Priego T, Martín AI, Villanúa MA, López-Calderón A. Anti-inflammatory effect of the ghrelin agonist growth hormone-releasing peptide-2 (GHRP-2) in arthritic rats. Am J Physiol Endocrinol Metab. 2005;288(3):E486–92. 10.1152/ajpendo.00196.2004. 15507538 10.1152/ajpendo.00196.2004

[CR77] Gromada J, Franklin I, Wollheim CB. Alpha-cells of the endocrine pancreas: 35 years of research but the enigma remains. Endocr Rev. 2007;28(1):84–116. 10.1210/er.2006-0007. 17261637 10.1210/er.2006-0007

[CR78] Gromotowicz-Poplawska A, Stankiewicz A, Kramkowski K, Gradzka A, Wojewodzka-Zelezniakowicz M, Dzieciol J, Szemraj J, Chabielska E. The acute prothrombotic effect of aldosterone in rats is partially mediated via angiotensin II receptor type 1. Thromb Res. 2016;138:114–20. 26709040 10.1016/j.thromres.2015.12.008

[CR79] Gunst J, Debaveye Y, Güiza F, Dubois J, Bruyn AD, Dauwe D, et al. Tight blood-glucose control without early parenteral nutrition in the ICU. N Engl J Med. 2023;389(13):1180–90. 37754283 10.1056/NEJMoa2304855

[CR80] Guo RF, Ward PA. Role of C5a in inflammatory responses. Annu Rev Immunol. 2005;23:821–52. 10.1146/annurev.immunol.23.021704.115835. 15771587 10.1146/annurev.immunol.23.021704.115835

[CR81] Guo H, Calkins JH, Sigel MM, Lin T. Interleukin-2 is a potent inhibitor of Leydig cell steroidogenesis. Endocrinology. 1990;127(3):1234–9. 10.1210/endo-127-3-1234. 2167211 10.1210/endo-127-3-1234

[CR82] Hahn PY, Wang P, Tait SM, Ba ZF, Reich SS, Chaudry IH. Sustained elevation in circulating catecholamine levels during polymicrobial sepsis. Shock. 1995;4(4):269–73. 8564555 10.1097/00024382-199510000-00007

[CR83] Hancock ML, Meyer RC, Mistry M, et al. Insulin receptor associates with promoters genome-wide and regulates gene expression. Cell. 2019;177(3):722-36.e22. 30955890 10.1016/j.cell.2019.02.030 PMC6478446

[CR84] Hansen TK, Thiel S, Wouters PJ, Christiansen JS, Van den Berghe G. Intensive insulin therapy exerts antiinflammatory effects in critically ill patients and counteracts the adverse effect of low mannose-binding lectin levels. J Clin Endocrinol Metab. 2003;88(3):1082–8. 10.1210/jc.2002-021478. 12629088 10.1210/jc.2002-021478

[CR85] Hartmann C, Radermacher P, Wepler M. NußbaumB non-hemodynamic effects of catecholamines. Shock. 2017;48(4):390–400. 28915214 10.1097/SHK.0000000000000879

[CR86] Hasselgren P, Talamini M, James JH, Fischer JE. Protein metabolism in different types of skeletal muscle during early and late sepsis in rats. Arch Surg. 1986;121(8):918–23. 3729710 10.1001/archsurg.1986.01400080064011

[CR87] Hasselgren PO, James JH, Benson DW, Hall-Angerås M, Angerås U, Hiyama DT, Li S, Fischer JE. Total and myofibrillar protein breakdown in different types of rat skeletal muscle: effects of sepsis and regulation by insulin. Metabolism. 1989;38(7):634–40. 10.1016/0026-0495(89)90100-5. 2661965 10.1016/0026-0495(89)90100-5

[CR88] Heffner AC, Horton JM, Marchick MR, Jones AE. Etiology of illness in patients with severe sepsis admitted to the hospital from the emergency department. Clin Infect Dis. 2010;50(6):814–20. 10.1086/650580. 20144044 10.1086/650580 PMC2921279

[CR89] Hofmaenner DA, Kleyman A, Press A, Bauer M, Singer M. The many roles of cholesterol in sepsis: a review. Am J Respir Crit Care Med. 2021;205:388–96. 10.1164/rccm.202105-1197TR PMC8886946 34715007

[CR90] Holst JJ, Wewer Albrechtsen NJ, Pedersen J, Knop FK. Glucagon and amino acids are linked in a mutual feedback cycle: the liver-α-cell axis. Diabetes. 2017;66(2):235. 10.2337/db16-0994. 28108603 10.2337/db16-0994

[CR91] Hong J, Zhang L, Lai Y, Chen X, Chen Y, Yang J. Causal association between thyroid dysfunction and sepsis: a two-sample mendelian randomization study. Front Endocrinol. 2024;15:1348248. 10.3389/fendo.2024.1348248. 10.3389/fendo.2024.1348248 PMC10995304 38586450

[CR92] Hoshino E, Pichard C, Greenwood CE, Kuo GC, Cameron RG, Kurian R, Kearns JP, Allard JP, Jeejeebhoy KN. Body composition and metabolic rate in rat during a continuous infusion of cachectin. Am J Physiol. 1991;260(1 Pt 1):E27-36. 10.1152/ajpendo.1991.260.1.E27. 1987791 10.1152/ajpendo.1991.260.1.E27

[CR93] Hotchkiss RS, Moldawer LL, Opal SM, Reinhart K, Turnbull IR, Vincent JL. Sepsis and septic shock. Nat Rev Dis Primers. 2016;2:16045. 10.1038/nrdp.2016.45. 28117397 10.1038/nrdp.2016.45 PMC5538252

[CR94] Hsieh M-S, How C-K, Hsieh VC-R, Chen P-C. Preadmission antihypertensive drug use and sepsis outcome: impact of angiotensin-converting enzyme inhibitors (ACEIs) and angiotensin receptor blockers (ARBs). Shock. 2020;53(4):407–15. 31135703 10.1097/SHK.0000000000001382

[CR95] Hsuchou H, Pan W, Kastin AJ. The fasting polypeptide FGF21 can enter brain from blood. Peptides. 2007;28:2382–6. 10.1016/j.peptides.2007.10.007. 17996984 10.1016/j.peptides.2007.10.007 PMC2151924

[CR96] Hummel RP 3rd, James JH, Warner BW, Hasselgren PO, Fischer JE. Evidence that cathepsin B contributes to skeletal muscle protein breakdown during sepsis. Arch Surg. 1988;123(2):221–4. 10.1001/archsurg.1988.01400260105013. 3341905 10.1001/archsurg.1988.01400260105013

[CR97] Inagaki T, Dutchak P, Zhao G, Ding X, Gautron L, Parameswara V, et al. Endocrine regulation of the fasting response by PPARα-mediated induction of fibroblast growth factor 21. Cell Metab. 2007;5:415–25. 10.1016/j.cmet.2007.05.003. 17550777 10.1016/j.cmet.2007.05.003

[CR98] Inagaki T, Lin VY, Goetz R, Mohammadi M, Mangelsdorf DJ, Kliewer SA. Inhibition of growth hormone signaling by the fasting-induced hormone FGF21. Cell Metab. 2008;8:77–83. 10.1016/j.cmet.2008.05.006. 18585098 10.1016/j.cmet.2008.05.006 PMC2575072

[CR99] Ingels C, Gunst J, Van den Berghe G. Endocrine and metabolic alterations in sepsis and implications for treatment. Crit Care Clin. 2018;34(1):81–96. 10.1016/j.ccc.2017.08.006. 29149943 10.1016/j.ccc.2017.08.006

[CR100] Jadhav AP, Sadaka FG. Angiotensin II in septic shock. Am J Emerg Med. 2019;37(6):1169–74. 30935784 10.1016/j.ajem.2019.03.026

[CR101] Jepson MM, Pell JM, Bates PC, Millward DJ. The effects of endotoxaemia on protein metabolism in skeletal muscle and liver of fed and fasted rats. Biochem J. 1986;235(2):329–36. 10.1042/bj2350329. 3527153 10.1042/bj2350329 PMC1146691

[CR102] Jeschke MG, Einspanier R, Klein D, Jauch KW. Insulin attenuates the systemic inflammatory response to thermal trauma. Mol Med. 2002;8(8):443–50. 12435855 PMC2040014

[CR103] Jeschke MG, Klein D, Bolder U, Einspanier R. Insulin attenuates the systemic inflammatory response in endotoxemic rats. Endocrinology. 2004;145(9):4084–93. 10.1210/en.2004-0592. 15192048 10.1210/en.2004-0592

[CR104] Jeschke MG, Klein D, Herndon DN. Insulin treatment improves the systemic inflammatory reaction to severe trauma. Ann Surg. 2004;239(4):553–60. 10.1097/01.sla.0000118569.10289. 15024317 10.1097/01.sla.0000118569.10289.ad PMC1356261

[CR105] Jeschke MG, Rensing H, Klein D, et al. Insulin prevents liver damage and preserves liver function in lipopolysaccharide-induced endotoxemic rats. J Hepatol. 2005;42(6):870–9. 10.1016/j.jhep.2004.12.036. 15885358 10.1016/j.jhep.2004.12.036

[CR106] Johnson CL, Weston JY, Chadi SA, Fazio EN, Huff MW, Kharitonenkov A, et al. Fibroblast growth factor 21 reduces the severity of cerulein-induced pancreatitis in mice. Gastroenterology. 2009;137:1795–804. 10.1053/j.gastro.2009.07.064. 19664632 10.1053/j.gastro.2009.07.064

[CR107] Jung WJ, Park BH, Chung KS, et al. Glucagon levels, disease severity, and outcome in severe sepsis. Shock. 2015;43(6):563–8. 10.1097/SHK.0000000000000344. 25978809 10.1097/SHK.0000000000000344

[CR108] Jurasinski CV, Kilpatrick L, Vary TC. Amrinone prevents muscle protein wasting during chronic sepsis. Am J Physiol. 1995;268(3 Pt 1):E491-500. 10.1152/ajpendo.1995.268.3.E491. 7534991 10.1152/ajpendo.1995.268.3.E491

[CR109] Kayali AG, Young VR, Goodman MN. Sensitivity of myofibrillar proteins to glucocorticoid-induced muscle proteolysis. Am J Physiol. 1987;252(5 Pt 1):E621–6. 10.1152/ajpendo.1987.252.5.E621. 3578511 10.1152/ajpendo.1987.252.5.E621

[CR110] Kelly FJ, Goldspink DF. The differing responses of four muscle types to dexamethasone treatment in the rat. Biochem J. 1982;208(1):147–51. 10.1042/bj2080147. 6186244 10.1042/bj2080147 PMC1153940

[CR111] Khardori R, Castillo D. Endocrine and metabolic changes during sepsis: an update. Med Clin North Am. 2012;96(6):1095–105. 10.1016/j.mcna.2012.09.005. 23102479 10.1016/j.mcna.2012.09.005

[CR112] Kharitonenkov A, Shiyanova TL, Koester A, Ford AM, Micanovic R, Galbreath EJ, et al. FGF-21 as a novel metabolic regulator. J Clin Invest. 2005;115:1627–35. 10.1172/JCI23606. 15902306 10.1172/JCI23606 PMC1088017

[CR113] Kim J, Kim YA, Hwangbo B, et al. Effect of antihypertensive medications on sepsis-related outcomes: a population-based cohort study. Crit Care Med. 2019;47(5):e386–93. 30688717 10.1097/CCM.0000000000003654

[CR114] Klune JR, Dhupar R, Cardinal J, Billiar TR, Tsung A. HMGB1: endogenous danger signaling. Mol Med. 2008;14(7–8):476–84. 10.2119/2008-00034.klune. 18431461 10.2119/2008-00034.Klune PMC2323334

[CR115] Kojima M, Kangawa K. Ghrelin: structure and function. Physiol Rev. 2005;85(2):495–522. 10.1152/physrev.00012.2004. 15788704 10.1152/physrev.00012.2004

[CR116] Koskinas J, Gomatos IP, Tiniakos DG, Memos N, Boutsikou M, Garatzioti A, Archimandritis A, Betrosian A. Liver histology in ICU patients dying from sepsis: a clinico-pathological study. S.l. World J Gastroenterol. 2008;14(9):1389–93. 18322953 10.3748/wjg.14.1389 PMC2693687

[CR117] Kothiwale VA, Patil P, Gaur S. Correlation of thyroid hormone profile with the acute physiology and chronic health evaluation II score as a prognostic marker in patients with sepsis in the intensive care unit. J Assoc Physicians India. 2018;66(7):59–62. 31325264

[CR118] La Via L, Sangiorgio G, Stefani S, et al. The global burden of sepsis and septic shock. Epidemiologia. 2024;5(3):456–78. 10.3390/epidemiologia5030032. 39189251 10.3390/epidemiologia5030032 PMC11348270

[CR119] Langouche L, Vander Perre S, Marques M, et al. Impact of early nutrient restriction during critical illness on the nonthyroidal illness syndrome and its relation with outcome: a randomized, controlled clinical study. J Clin Endocrinol Metab. 2013;98(3):1006–13. 10.1210/jc.2012-2809. 23348400 10.1210/jc.2012-2809

[CR120] Lee S, Farwell AP. Euthyroid Sick Syndrome. Compr Physiol. 2016;6(2):1071–80. 10.1002/cphy.c150017. 27065175 10.1002/cphy.c150017

[CR121] Leite-Moreira AF, Soares JB. Physiological, pathological and potential therapeutic roles of ghrelin. Drug Discovery Today. 2007;12(7–8):276–88. 10.1016/j.drudis.2007.02.009. 17395087 10.1016/j.drudis.2007.02.009

[CR122] Levy B. Lactate and shock state: the metabolic view. Curr Opin Crit Care. 2006;12(4):315–21. 10.1097/01.ccx.0000235208.77450.15. 16810041 10.1097/01.ccx.0000235208.77450.15

[CR123] Li L, Messina JL. Acute insulin resistance following injury. Trends Endocrinol Metab. 2009;20(9):429–35. 19800814 10.1016/j.tem.2009.06.004 PMC2939005

[CR124] Li X, Zhu Z, Zhou T, Cao X, Lu T, Liang Y, He J, Liu C, Dou Z, Shen B. Early increases in serum FGF21 levels predict mortality of septic patients. Cytokine. 2018;111:428–33. 10.1016/j.cyto.2018.05.020. 29861384 10.1016/j.cyto.2018.05.020

[CR125] Li X, Shen H, Zhou T, Cao X, Chen Y, Liang Y, Lu T, He J, Dou Z, Liu C, Tang Y, Zhu Z. Does an increase in serum FGF21 level predict 28-day mortality of critical patients with sepsis and ARDS? Respir Res. 2021. 10.1186/s12931-021-01778-w. 34154595 10.1186/s12931-021-01778-w PMC8216835

[CR126] Liang H, Song H, Zhai R, et al. Corticosteroids for treating sepsis in adult patients: a systematic review and meta-analysis. Front Immunol. 2021. 10.3389/fimmu.2021.709155. 34484209 10.3389/fimmu.2021.709155 PMC8415513

[CR127] Lim CF, Docter R, Visser TJ, et al. Inhibition of thyroxine transport into cultured rat hepatocytes by serum of nonuremic critically ill patients: effects of bilirubin and nonesterified fatty acids. J Clin Endocrinol Metab. 1993;76(5):1165–72. 10.1210/jcem.76.5.8496307. 8496307 10.1210/jcem.76.5.8496307

[CR128] Lin X, Shi S, Shi S. Sepsis leads to thyroid impairment and dysfunction in rat model. Tissue Cell. 2016;48(5):511–5. 10.1016/j.tice.2016.07.001. 27521250 10.1016/j.tice.2016.07.001

[CR129] Liu V, Escobar GJ, Greene JD, et al. Hospital deaths in patients with sepsis from 2 independent cohorts. JAMA. 2014;312:9092. 10.1001/jama.2014.5804 24838355

[CR130] Lu Z, Tao G, Sun X, Zhang Y, Jiang M, Liu Y, Ling M, Zhang J, Xiao W, Hua T, Zhu H, Yang M. Association of blood glucose level and glycemic variability with mortality in sepsis patients during ICU hospitalization. Front Public Health. 2022;29(10):857368. 10.3389/fpubh.2022.857368. 10.3389/fpubh.2022.857368 PMC9099235 35570924

[CR131] Lucas C, Christie GA, Waring WS. Rapid onset of haemodynamic effects after angiotensin converting enzyme-inhibitor overdose: implications for initial patient triage. Emerg Med J. 2006;23:854–7. 17057137 10.1136/emj.2006.038836 PMC2464381

[CR132] Lyte M. The role of catecholamines in Gram-negative sepsis. Med Hypotheses. 1992;37(4):255–8. 1625603 10.1016/0306-9877(92)90197-k

[CR133] Mantzoros CS. The role of leptin in human obesity and disease: a review of current evidence. Ann Intern Med. 1999;130(8):671–80. 10.7326/0003-4819-130-8-199904200-00014. 10215564 10.7326/0003-4819-130-8-199904200-00014

[CR134] Marik PE, Bellomo R. Stress hyperglycemia: an essential survival response!*. Crit Care. 2013;17(2):305. 10.1186/cc12514. 23470218 10.1186/cc12514 PMC3672537

[CR135] Marik PE, Raghavan M. Stress-hyperglycemia, insulin and immunomodulation in sepsis. Intensive Care Med. 2004;30(5):748–56. 14991101 10.1007/s00134-004-2167-y

[CR136] Marik PE, Pastores SM, Annane D, et al. Recommendations for the diagnosis and management of corticosteroid insufficiency in critically ill adult patients: consensus statements from an international task force by the American College of critical care medicine. Crit Care Med. 2008;36(6):1937–49. 10.1097/CCM.0b013e31817603ba. 18496365 10.1097/CCM.0b013e31817603ba

[CR137] Martin GS. Sepsis, severe sepsis and septic shock: changes in incidence, pathogens and outcomes. Expert Rev Anti Infect Ther. 2012;10:701706. 10.1586/eri.12.50 PMC3488423 22734959

[CR138] Martin GS, Mannino DM, Eaton S, et al. The epidemiology of sepsis in the United States from 1979 through 2000. N Engl J Med. 2003;348:15461554. 10.1056/NEJMoa022139 12700374

[CR139] Martín-Fernandez B, Rubio-Navarro A, Cortegano I, et al. Aldo- sterone induces renal fibrosis and inflammatory M1-macrophage subtype via mineralocorticoid receptor in rats. PLoS ONE. 2016;11(1): e0145946. 26730742 10.1371/journal.pone.0145946 PMC4701403

[CR140] Maruna P, Gürlich R, Frasko R, Rosicka M. Ghrelin and leptin elevation in postoperative intra-abdominal sepsis. Eur Surg Res. 2005;37(6):354–9. 10.1159/000090336. 16465060 10.1159/000090336

[CR141] Mebis L, Eerdekens A, Güiza F, et al. Contribution of nutritional deficit to the pathogenesis of the nonthyroidal illness syndrome in critical illness: a rabbit model study. Endocrinology. 2012;153(2):973–84. 10.1210/en.2011-1411. 22166982 10.1210/en.2011-1411

[CR142] Mechanick JI, Nierman DM. Gonadal steroids in critical illness. Crit Care Clin. 2006;22(1):87–vii. 10.1016/j.ccc.2005.08.005. 16399021 10.1016/j.ccc.2005.08.005

[CR143] Meng J, Li X, Xiao Y, et al. Intensive or liberal glucose control in intensive care units for septic patients? A meta-analysis of randomized controlled trials. Diabetes Metab Syndr. 2024;18(5):103045. 10.1016/j.dsx.2024.103045. 38796958 10.1016/j.dsx.2024.103045

[CR144] Meyer NJ, Prescott HC. Sepsis and septic shock. N Engl J Med. 2024;391:2133–46. 10.1056/NEJMra2403213. 39774315 10.1056/NEJMra2403213

[CR145] Michalaki M, Vagenakis AG, Makri M, Kalfarentzos F, Kyriazopoulou V. Dissociation of the early decline in serum T(3) concentration and serum IL-6 rise and TNFalpha in nonthyroidal illness syndrome induced by abdominal surgery. J Clin Endocrinol Metab. 2001;86(9):4198–205. 10.1210/jcem.86.9.7795. 11549650 10.1210/jcem.86.9.7795

[CR146] Michie HR. Metabolism of sepsis and multiple organ failure. World J Surg. 1996;20(4):460–4. 10.1007/s002689900072. 8662135 10.1007/s002689900072

[CR147] Miller RA, Birnbaum MJ. Glucagon: acute actions on hepatic metabolism. Diabetologia. 2016;59(7):1376–81. 10.1007/s00125-016-3955-y. 27115415 10.1007/s00125-016-3955-y

[CR148] Morrison DC, Ryan JL. Endotoxins and disease mechanisms. Annu Rev Med. 1987;38:417–32. 10.1146/annurev.me.38.020187.002221. 3555304 10.1146/annurev.me.38.020187.002221

[CR149] Muniz-Santos R, Lucieri-Costa G, Almeida MAP, Moraes-de-Souza I, Brito MASM, Silva AR, Gonc¸alves-de-Albuquerque CF. Lipid oxidation dysregulation: an emerging player in the pathophysiology of sepsis s.l. Front Immunol. 2023;14:1224335. 37600769 10.3389/fimmu.2023.1224335 PMC10435884

[CR150] Nasir N, Jamil B, Siddiqui S, Talat N, Khan FA, Hussain R. Mortality in sepsis and its relationship with gender. Pak J Med Sci. 2015;31(5):1201–6. 10.12669/pjms.315.6925. 26649014 10.12669/pjms.315.6925 PMC4641283

[CR151] Nematy M, O’Flynn JE, Wandrag L, et al. Changes in appetite related gut hormones in intensive care unit patients: a pilot cohort study. Crit Care. 2006;10(1):R10. 10.1186/cc3957. 16420657 10.1186/cc3957 PMC1550795

[CR152] NICE-SUGAR Study Investigators, Finfer S, Chittock DR, et al. Intensive versus conventional glucose control in critically ill patients. N Engl J Med. 2009;360(13):1283–97. 10.1056/NEJMoa0810625. 19318384 10.1056/NEJMoa0810625

[CR153] Nikitopoulou I, Kampisiouli E, Jahaj E, et al. Ghrelin alterations during experimental and human sepsis. Cytokine. 2020;127:154937. 10.1016/j.cyto.2019.154937. 31830702 10.1016/j.cyto.2019.154937

[CR154] Nishimura T, Nakatake Y, Konishi M, Itoh N. Identification of a novel FGF, FGF-21, preferentially expressed in the liver. Biochim Biophys Acta. 2000;1492:203–6. 10858549 10.1016/s0167-4781(00)00067-1

[CR155] Noritomi DT, et al. Metabolic acidosis in patients with severe sepsis and septic shock: a longitudinal quantitative study. Crit Care Med. 2009. 10.1097/ccm.0b013e3181a59165. 19885998 10.1097/ccm.0b013e3181a59165

[CR156] Onenli-Mungan N, Yildizdas D, Yapicioglu H, Topaloglu AK, Yüksel B, Ozer G. Growth hormone and insulin-like growth factor 1 levels and their relation to survival in children with bacterial sepsis and septic shock. J Paediatr Child Health. 2004;40(4):221–6. 10.1111/j.1440-1754.2004.00342.x. 15009554 10.1111/j.1440-1754.2004.00342.x

[CR157] Papastathi C, Mavrommatis A, Mentzelopoulos S, Konstandelou E, Alevizaki M, Zakynthinos S. Insulin-like Growth Factor I and its binding protein 3 in sepsis. Growth Horm IGF Res. 2013;23(4):98–104. 10.1016/j.ghir.2013.03.005. 23611528 10.1016/j.ghir.2013.03.005

[CR158] Papathanassoglou ED, Moynihan JA, Ackerman MH, Mantzoros CS. Serum leptin levels are higher but are not independently associated with severity or mortality in the multiple organ dysfunction/systemic inflammatory response syndrome: a matched case control and a longitudinal study. Clin Endocrinol. 2001;54(2):225–33. 10.1046/j.1365-2265.2001.01209.x. 10.1046/j.1365-2265.2001.01209.x 11207638

[CR159] Pastore L, Tessitore A, Martinotti S, Toniato E, Alesse E, Bravi MC, Ferri C, Desideri G, Gulino A, Santucci A. Angiotensin II stimulates intercellular adhesion molecule-1 (ICAM-1) expression by human vascular endothelial cells and increases soluble ICAM-1 release in vivo. Circulation. 1999;100:1646–52. 10517737 10.1161/01.cir.100.15.1646

[CR160] Patel R, Bookout AL, Magomedova L, Owen BM, Consiglio GP, Shimizu M, et al. Glucocorticoids regulate the metabolic hormone FGF21 in a feed-forward loop. Mol Endocrinol. 2015;29:213–23. 10.1210/me.2014-1259. 25495872 10.1210/me.2014-1259 PMC4318881

[CR161] Peerapornratana S, Manrique-Caballero CL, Gomez H, Kellum JA. Acute kidney injury from sepsis: current concepts, epidemiology, pathophysiology, prevention and treatment. Kidney Int. 2019;96(5):1083–99. 31443997 10.1016/j.kint.2019.05.026 PMC6920048

[CR162] Peeters RP, Wouters PJ, Kaptein E, van Toor H, Visser TJ, Van den Berghe G. Reduced activation and increased inactivation of thyroid hormone in tissues of critically ill patients. J Clin Endocrinol Metab. 2003;88(7):3202–11. 10.1210/jc.2002-022013. 12843166 10.1210/jc.2002-022013

[CR163] Rababa M, Bani Hamad D, Hayajneh AA. Sepsis assessment and management in critically ill adults: a systematic review. PLoS ONE. 2022. 10.1371/journal.pone.0270711. 35776738 10.1371/journal.pone.0270711 PMC9249173

[CR164] Rannels SR, Jefferson LS. Effects of glucocorticoids on muscle protein turnover in perfused rat hemicorpus. Am J Physiol. 1980;238(6):E564–72. 10.1152/ajpendo.1980.238.6.E564. 6155787 10.1152/ajpendo.1980.238.6.E564

[CR165] Read JL, Cheng EY. Intensive insulin therapy for acute hyperglycemia. AACN Adv Crit Care. 2007;18(2):200–12. 17473549 10.1097/01.AACN.0000269264.22041.1c

[CR166] Rhodes A, Evans LE, Alhazzani W, et al. Surviving sepsis campaign: international guidelines for management of sepsis and septic shock: 2016. Intensive Care Med. 2017;43(3):304–77. 10.1007/s00134-017-4683-6. 28101605 10.1007/s00134-017-4683-6

[CR167] Rittirsch D, Flierl MA, Ward PA. Harmful molecular mechanisms in sepsis. Nat Rev Immunol. 2008;8(10):776–87. 18802444 10.1038/nri2402 PMC2786961

[CR168] Rodriguez-Perez A, Palos-Paz F, Kaptein E, et al. Identification of molecular mechanisms related to nonthyroidal illness syndrome in skeletal muscle and adipose tissue from patients with septic shock. Clin Endocrinol. 2008;68(5):821–7. 10.1111/j.1365-2265.2007.03102.x. 10.1111/j.1365-2265.2007.03102.x 17986277

[CR169] Rossi MA, Celes MR, Prado CM, Saggioro FP. Myocardial structural changes in long-term human severe sepsis/septic shock may be responsible for cardiac dysfunction s.l. Shock. Heart Lung Circ. 2007. 10.1016/j.hlc.2007.11.095. 10.1097/01.shk.0000235141.05528.47 17172974

[CR170] Rudiger A, Singer M. Decatecholaminisation during sepsis. Crit Care. 2016;20(1):309. 27716402 10.1186/s13054-016-1488-x PMC5048664

[CR171] Ruff RL, Secrist D. Inhibitors of prostaglandin synthesis or cathepsin B prevent muscle wasting due to sepsis in the rat. J Clin Invest. 1984;73(5):1483–6. 10.1172/JCI111352.PMID:6715547. 6715547 10.1172/JCI111352 PMC425171

[CR172] Ruiz-Ortega M, Ruperez M, Lorenzo O, Esteban V, Blanco J, Mezzano S, Egido J. Angiotensin II regulates the synthesis of proinflammatory cytokines and chemokines in the kidney. Kidney Int Suppl. 2002;62:S12–22. 10.1046/j.1523-1755.62.s82.4.x 12410849

[CR173] Sachse A, Wolf G. Angiotensin II-induced reactive oxygen species and the kidney. J Am Soc Nephrol. 2007;18:2439–46. 17687073 10.1681/ASN.2007020149

[CR174] Saia RS, Garcia FM, Cárnio EC. Estradiol protects female rats against sepsis induced by Enterococcus faecalis improving leukocyte bactericidal activity. Steroids. 2015;102:17–26. 10.1016/j.steroids.2015.06.016. 26143494 10.1016/j.steroids.2015.06.016

[CR175] Schlapbach LJ, Watson RS, Sorce LR, et al. International consensus criteria for pediatric sepsis and septic shock. JAMA. 2024;331(8):665–74. 10.1001/jama.2024.0179. 38245889 10.1001/jama.2024.0179 PMC10900966

[CR176] Schuetz P, Müller B. The hypothalamic-pituitary-adrenal axis in critical illness. Endocrinol Metab Clin North Am. 2006;35(4):823–38. 10.1016/j.ecl.2006.09.013. 17127149 10.1016/j.ecl.2006.09.013

[CR177] Schulte W, Bernhagen J, Bucala R. Cytokines in sepsis: potent immunoregulators and potential therapeutic targets–an updated view. Mediators Inflamm. 2013;2013:165974. 10.1155/2013/165974. 23853427 10.1155/2013/165974 PMC3703895

[CR178] Seim I, Collet C, Herington AC, Chopin LK. Revised genomic structure of the human ghrelin gene and identification of novel exons, alternative splice variants and natural antisense transcripts. BMC Genom. 2007;8:298. 10.1186/1471-2164-8-298. 10.1186/1471-2164-8-298 PMC2014779 17727735

[CR179] Selvaraj N, Bobby Z, Sridhar MG. Is euthyroid sick syndrome a defensive mechanism against oxidative stress? Med Hypotheses. 2008;71(3):404–5. 10.1016/j.mehy.2007.11.019. 18524495 10.1016/j.mehy.2007.11.019

[CR180] Senchenkova EY, Russell J, Almeida-Paula LD, Harding JW, Granger DN. Angiotensin II-mediated microvascular thrombosis. Hypertension. 2010;56:1089–95. 20975035 10.1161/HYPERTENSIONAHA.110.158220 PMC3023299

[CR181] Shapiro NI, Douglas IS, Brower RG, Brown SM, Exline MC, Ginde AA, et al. Early restrictive or liberal fluid management for sepsis-induced hypotension. N Engl J Med. 2023;388(6):499–510. 10.1056/NEJMoa2212663. 36688507 10.1056/NEJMoa2212663 PMC10685906

[CR182] Shepard CC, Levy L, Fasal P. Further experience with the rapid bactericidal effect of rifampin on Mycobacterium leprae. Am J Trop Med Hyg. 1974;23(6):1120–4. 10.4269/ajtmh.1974.23.1120. 4611256 10.4269/ajtmh.1974.23.1120

[CR183] Sibilia V, Pagani F, Mrak E, Dieci E, Tulipano G, Ferrucci F. Pharmacological characterization of the ghrelin receptor mediating its inhibitory action on inflammatory pain in rats. Amino Acids. 2012;43(4):1751–9. 10.1007/s00726-012-1260-8. 22407485 10.1007/s00726-012-1260-8 PMC3448055

[CR184] Singer M, Deutschman CS, Seymour CW, et al. The third international consensus definitions for sepsis and septic shock (Sepsis-3). JAMA. 2016;315(8):801–10. 10.1001/jama.2016.0287. 26903338 10.1001/jama.2016.0287 PMC4968574

[CR185] Song F, Zhong LJ, Han L, et al. Intensive insulin therapy for septic patients: a meta-analysis of randomized controlled trials. Biomed Res Int. 2014;2014:698265. 10.1155/2014/698265. 25136614 10.1155/2014/698265 PMC4086473

[CR186] Sorimachi H, Suzuki K. Sequence comparison among muscle-specific calpain, p94, and calpain subunits. Biochim Biophys Acta. 1992;1160(1):55–62. 10.1016/0167-4838(92)90037-e. 1420333 10.1016/0167-4838(92)90037-e

[CR187] Spratt DI. Altered gonadal steroidogenesis in critical illness: is treatment with anabolic steroids indicated? Best Pract Res Clin Endocrinol Metab. 2001;15(4):479–94. 10.1053/beem.2001.0165. 11800519 10.1053/beem.2001.0165

[CR188] Takebayashi K, Aso Y, Inukai T. Initiation of insulin therapy reduces serum concentrations of high-sensitivity C-reactive protein in patients with type 2 diabetes. Metabolism. 2004;53(6):693–9. 10.1016/j.metabol.2004.01.003. 15164314 10.1016/j.metabol.2004.01.003

[CR189] Taylor JH, Beilman GJ. Hyperglycemia in the intensive care unit: no longer just a marker of illness severity. Surg Infect. 2005;6(2):233–45. 10.1089/sur.2005.6.233. 10.1089/sur.2005.6.233 16128630

[CR190] Téblick A, Vander Perre S, Pauwels L, et al. The role of pro-opiomelanocortin in the ACTH-cortisol dissociation of sepsis. Crit Care. 2021;25(1):65. 10.1186/s13054-021-03475-y. 33593393 10.1186/s13054-021-03475-y PMC7885358

[CR191] Téblick A, De Bruyn L, Van Oudenhove T, et al. Impact of hydrocortisone and of CRH infusion on the hypothalamus-pituitary-adrenocortical axis of septic male mice. Endocrinology. 2022;163(1):bqab222. 10.1210/endocr/bqab222. 34698826 10.1210/endocr/bqab222 PMC8599906

[CR192] Theil MM, Miyake S, Mizuno M, et al. Suppression of experimental autoimmune encephalomyelitis by ghrelin. J Immunol. 2009;183(4):2859–66. 10.4049/jimmunol.0803362. 19620309 10.4049/jimmunol.0803362

[CR193] Thurston AJ. Of blood, inflammation and gunshot wounds: the history of the control of sepsis. Aust N Z J Surg. 2000;70(12):855–61. 10.1046/j.1440-1622.2000.01983.x. 11167573 10.1046/j.1440-1622.2000.01983.x

[CR194] Tiao G, Fagan JM, Samuels N, James JH, Hudson K, Lieberman M, Fischer JE, Hasselgren PO. Sepsis stimulates nonlysosomal, energy-dependent proteolysis and increases ubiquitin mRNA levels in rat skeletal muscle. J Clin Invest. 1994;94(6):2255–64. 10.1172/JCI117588. 7989581 10.1172/JCI117588 PMC330052

[CR195] Timmins AC, Cotterill AM, Hughes SC, et al. Critical illness is associated with low circulating concentrations of insulin-like growth factors-I and -II, alterations in insulin-like growth factor binding proteins, and induction of an insulin-like growth factor binding protein 3 protease. Crit Care Med. 1996;24(9):1460–6. 10.1097/00003246-199609000-00006. 8797616 10.1097/00003246-199609000-00006

[CR196] Tracey KJ, Morgello S, Koplin B, Fahey TJ 3rd, Fox J, Aledo A, Manogue KR, Cerami A. Metabolic effects of cachectin/tumor necrosis factor are modified by site of production. Cachectin/tumor necrosis factor-secreting tumor in skeletal muscle induces chronic cachexia, while implantation in brain induces predominantly acute anorexia. J Clin Invest. 1990;86(6):2014–24. 10.1172/JCI114937. 2254457 10.1172/JCI114937 PMC329839

[CR197] Tsuchimochi W, Kyoraku I, Yamaguchi H, et al. Ghrelin prevents the development of experimental diabetic neuropathy in rodents. Eur J Pharmacol. 2013;702(1–3):187–93. 10.1016/j.ejphar.2013.01.035. 23396232 10.1016/j.ejphar.2013.01.035

[CR198] Turnbull AV, Rivier CL. Regulation of the hypothalamic-pituitary-adrenal axis by cytokines: actions and mechanisms of action. Physiol Rev. 1999;79(1):1–71. 10.1152/physrev.1999.79.1.1. 9922367 10.1152/physrev.1999.79.1.1

[CR199] Tzanela M, Orfanos SE, Tsirantonaki M, et al. Leptin alterations in the course of sepsis in humans. In Vivo. 2006;20(4):565–70. 16900791

[CR200] Uchino S, Kellum JA, Bellomo R, Doig GS, Morimatsu H, Morgera S, Schetz M, Tan I, Bouman C, Macedo E, et al. Acute renal failure in critically ill patients: a multinational, multicenter study. JAMA. 2005;294:813–8. 16106006 10.1001/jama.294.7.813

[CR201] Umpierrez GE, Isaacs SD, Bazargan N, You X, Thaler LM, Kitabchi AE. Hyperglycemia: an independent marker of in-hospital mortality in patients with undiagnosed diabetes. J Clin Endocrinol Metab. 2002;87(3):978–82. 10.1210/jcem.87.3.8341. 11889147 10.1210/jcem.87.3.8341

[CR202] Unger RH. Glucagon physiology and pathophysiology in the light of new advances. Diabetologia. 1985;28(8):574–8. 10.1007/BF00281991. 3902546 10.1007/BF00281991

[CR203] Van den Berghe GH. Acute and prolonged critical illness are two distinct neuroendocrine paradigms. Verh K Acad Geneeskd Belg. 1998a;60(6):487–520. 10230322

[CR204] Van den Berghe G. Leptin levels in protracted critical illness: effects of growth hormone-secretagogues and thyrotropin-releasing hormone. J Clin Endocrinol Metab. 1998b;83(9):3062–70. 10.1210/jc.83.9.306. 9745404 10.1210/jcem.83.9.5120

[CR205] Van den Berghe G. Dynamic neuroendocrine responses to critical illness. Front Neuroendocrinol. 2002;23(4):370–91. 10.1016/s0091-3022(02)00006-7. 12381331 10.1016/s0091-3022(02)00006-7

[CR206] Van den Berghe GH. Role of intravenous insulin therapy in critically ill patients. Endocr Pract. 2004;10(Suppl 2):17–20. 10.4158/EP.10.S2.17. 15251636 10.4158/EP.10.S2.17

[CR207] Van den Berghe G. Non-thyroidal illness in the ICU: a syndrome with different faces. Thyroid. 2014;24(10):1456–65. 10.1089/thy.2014.0202. 24845024 10.1089/thy.2014.0201 PMC4195234

[CR208] Van den Berghe G, Wouters P, Weekers F, et al. Intensive insulin therapy in critically ill patients. N Engl J Med. 2001a;345(19):1359–67. 10.1056/NEJMoa011300. 11794168 10.1056/NEJMoa011300

[CR209] Van den Berghe G, Wouters PJ, Bouillon R, Weekers F, Verwaest C, Schetz M, et al. Outcome benefit of intensive insulin therapy in the critically ill: insulin dose versus glycemic control. Crit Care Med. 2003;31(2):359–66. 12576937 10.1097/01.CCM.0000045568.12881.10

[CR210] Van den Berghe G, Wilmer A, Hermans G, Meersseman W, Wouters PJ, Milants I, et al. Intensive insulin therapy in the medical ICU. N Engl J Med. 2006;354(5):449–61. 16452557 10.1056/NEJMoa052521

[CR211] van den Berghe G, Weekers F, Baxter RC, et al. Five-day pulsatile gonadotropin-releasing hormone administration unveils combined hypothalamic-pituitary-gonadal defects underlying profound hypoandrogenism in men with prolonged critical illness. J Clin Endocrinol Metab. 2001b;86(7):3217–26. 10.1210/jcem.86.7.7680. 11443192 10.1210/jcem.86.7.7680

[CR212] van der Crabben SN, Blumer RM, Stegenga ME, et al. Early endotoxemia increases peripheral and hepatic insulin sensitivity in healthy humans. J Clin Endocrinol Metab. 2009;94(2):463–8. 18984669 10.1210/jc.2008-0761

[CR213] Vanhorebeek I, De Vos R, Mesotten D, et al. Protection of hepatocyte mitochondrial ultrastructure and function by strict blood glucose control with insulin in critically ill patients. Lancet. 2005;365(9453):53–9. 15639679 10.1016/S0140-6736(04)17665-4

[CR214] Vanwijngaerden YM, Wauters J, Langouche L, et al. Critical illness evokes elevated circulating bile acids related to altered hepatic transporter and nuclear receptor expression. Hepatology. 2011;54(5):1741–52. 10.1002/hep.24582. 21800341 10.1002/hep.24582

[CR215] Vary TC, Kimball SR. Sepsis-induced changes in protein synthesis: differential effects on fast- and slow-twitch muscles. Am J Physiol. 1992;262(6 Pt 1):C1513–9. 10.1152/ajpcell.1992.262.6.C1513. 1377447 10.1152/ajpcell.1992.262.6.C1513

[CR216] Vary TC, Voisin L, Cooney RN. Regulation of peptide-chain initiation in muscle during sepsis by interleukin-1 receptor antagonist. Am J Physiol. 1996;271(3 Pt 1):E513–20. 10.1152/ajpendo.1996.271.3.E513. 8843745 10.1152/ajpendo.1996.271.3.E513

[CR217] Virkamaki A, Yki-Jarvinen H. Mechanisms of insulin resistance during acute endotoxemia. Endocrinology. 1994;134(5):2072–8. 8156907 10.1210/endo.134.5.8156907

[CR218] Vlasselaers D, Milants I, Desmet L, et al. Intensive insulin therapy for patients in paediatric intensive care: a prospective, randomised controlled study. Lancet. 2009;373(9663):547–56. 10.1016/S0140-6736(09)60044-1. 19176240 10.1016/S0140-6736(09)60044-1

[CR219] Wang ZK, Xu LS, Wang SL, Liu LY, Hu XY, Zhu ZZ, et al. Influence of glucose– insulin–potassium on the levels of inflammatory cytokines and prognosis of MODS in the scalded rats. Chin J Burns. 2005;21(6):422–5. 16480620

[CR220] Wang YC, Gao F, Jia CY, Zhang WF, Tang CW, Hu DH. Regulating effects of different doses of insulin on serum IL-1 13 and IL-10 in early stage in severely scalded rats. J Fourth Mil Med Univ. 2006;27(22):2043–6.

[CR221] Wang Y, Xu GG, Wu JH, Zhao X, Miao Q, Chen W. Effect of intensive insulin therapy on serum proinflammatory cytokine levels in patients with systemic inflammatory response syndrome. Clin Educ Gen Pract. 2007;5(1):36–8.

[CR222] Warzecha Z, Ceranowicz P, Dembinski A, et al. Therapeutic effect of ghrelin in the course of cerulein-induced acute pancreatitis in rats. J Physiol Pharmacol. 2010;61(4):419–27. 20814069

[CR223] Wasyluk W, Zwolak A. Metabolic alterations in sepsis. J Clin Med. 2021;10:2412. 34072402 10.3390/jcm10112412 PMC8197843

[CR224] Wasyluk W, Zwolak A. Metabolic alterations in sepsis. J Clin Med. 2021;10(11):2412. 10.3390/jcm10112412. 34072402 10.3390/jcm10112412 PMC8197843

[CR225] Wasyluk W, Wasyluk M, Zwolak A. Sepsis as a pan-endocrine illness-endocrine disorders in septic patients. J Clin Med. 2021;10(10):2075. 10.3390/jcm10102075. 34066289 10.3390/jcm10102075 PMC8152097

[CR226] Wolf G, Wenzel U, Burns KD, Harris RC, Stahl RA, Thaiss F. Angiotensin II activates nuclear transcription factor-kappaB through AT1 and AT2 receptors. Kidney Int. 2002;61:1986–95. 12028439 10.1046/j.1523-1755.2002.00365.x

[CR227] Xu J, Tong L, Yao J, et al. Association of sex with clinical outcome in critically Ill sepsis patients: a retrospective analysis of the large clinical database MIMIC-III. Shock. 2019;52(2):146–51. 10.1097/SHK.0000000000001253. 30138298 10.1097/SHK.0000000000001253 PMC6687414

[CR228] Yamada T, Shojima N, Noma H, Yamauchi T, Kadowaki T. Glycemic control, mortality, and hypoglycemia in critically ill patients: a systematic review and network meta-analysis of randomized controlled trials. Intensive Care Med. 2017;43(1):1–15. 10.1007/s00134-016-4523-0. 27637719 10.1007/s00134-016-4523-0

[CR229] Yan F, Yuan L, Yang F, Wu G, Jiang X. Emerging roles of fibroblast growth factor 21 in critical disease. Front Cardiovasc Med. 2022. 10.3389/fcvm.2022.1053997. 36440004 10.3389/fcvm.2022.1053997 PMC9684205

[CR230] Yanni GN, Destariani CP, Lubis AN, Deliana M. Thyroid Hormone profile in children with sepsis: does euthyroid sick syndrome exist? Open Access Maced J Med Sci. 2019;7(7):1110–3. 10.3889/oamjms.2019.262. 31049090 10.3889/oamjms.2019.262 PMC6490479

[CR231] Yaribeygi H, Farrokhi FR, Butler AE, et al. Insulin resistance: review of the underlying molecular mechanisms. J Cell Physiol. 2019;234(6):8152–61. 30317615 10.1002/jcp.27603

[CR232] Yen PM. Physiological and molecular basis of thyroid hormone action. Physiol Rev. 2001;81(3):1097–142. 10.1152/physrev.2001.81.3.1097. 11427693 10.1152/physrev.2001.81.3.1097

[CR233] Yildizdaş D, Onenli-Mungan N, Yapicioğlu H, Topaloğlu AK, Sertdemir Y, Yüksel B. Thyroid hormone levels and their relationship to survival in children with bacterial sepsis and septic shock. J Pediatr Endocrinol Metab. 2004;17(10):1435–42. 15526723

[CR234] Zager RA, Johnson ACM, Hanson SY. Renal tubular triglyceride accumulation following endotoxic, toxic, and ischemic injury. s.l. Kidney Int. 2005;67(1):111–21. 15610234 10.1111/j.1523-1755.2005.00061.x

[CR235] Zamir O, Hasselgren PO, O’Brien W, Thompson RC, Fischer JE. Muscle protein breakdown during endotoxemia in rats and after treatment with interleukin-1 receptor antagonist (IL-1ra). Ann Surg. 1992;216(3):381–5. 10.1097/00000658-199209000-00018. 1417187 10.1097/00000658-199209000-00018 PMC1242630

[CR236] Zamir O, Hasselgren PO, Kunkel SL, Frederick J, Higashiguchi T, Fischer JE. Evidence that tumor necrosis factor participates in the regulation of muscle proteolysis during sepsis. Arch Surg. 1992;127(2):170–4. 10.1001/archsurg.1992.01420020052008. 1540094 10.1001/archsurg.1992.01420020052008

[CR237] Zhang X, Yeung DCY, Karpisek M, Stejskal D, Zhou Z-G, Liu F, et al. Serum FGF21 levels are increased in obesity and are independently associated with the metabolic syndrome in humans. Diabetes Metab Res Rev. 2008;57:1246–53. 10.2337/db07-1476. 10.2337/db07-1476 18252893

[CR238] Zhang F, Liu H, Liu D, Liu Y, Li H, Tan X, Liu F, Peng Y, Zhang H. Effects of RAAS inhibitors in patients with kidney disease. Curr Hypertens Rep. 2017;19:72. 28791529 10.1007/s11906-017-0771-9

[CR239] Zhang P, Pan S, Yuan S, Shang Y, Shu H. Abnormal glucose metabolism in virus associated sepsis. Front Cell Infect Microbiol. 2023;13:1120769. 10.3389/fcimb.2023.1120769. 37124033 10.3389/fcimb.2023.1120769 PMC10130199

[CR240] Zhang L, Tan R, Pan T, Qu H. Impact of thyroid hormones on predicting the occurrence of persistent inflammation, immunosuppression, and catabolism syndrome in patients with sepsis. Front Endocrinol. 2024;15:1417846. 10.3389/fendo.2024.1417846. 10.3389/fendo.2024.1417846 PMC11521835 39479266

[CR241] Zhao XD, Yao YM, Ma JX, Liang HP, Yan RM, Zhang LY, et al. Effects of intensive insulin therapy on serum immunoglobulin, complement levels and phagocytosis of monocytes in patients with severe trauma. Chin Crit Care Med. 2007a;19(5):279–82. 17490567

[CR242] Zhao XD, Liu B, Yao YM, Zhang LY, Liang HP. Effects of intensive insulin therapy at the early stage on IFN-γ and IL-18 in serum for severe trauma patients. Chin J Crit Care Med. 2007b;27(1):1–3.

[CR243] Zhou L, He R, Fang P, et al. Hepatitis B virus rigs the cellular metabolome to avoid innate immune recognition. Nat Commun. 2021;12(1):98. 10.1038/s41467-020-20316-8. 33397935 10.1038/s41467-020-20316-8 PMC7782485

